# Microbial Allies in the Olive Canopy: Endophyte Composition, Drivers, and their Role in Plant Protection

**DOI:** 10.1007/s00248-025-02676-0

**Published:** 2025-12-12

**Authors:** Dalila Crucitti, Francesco Carimi, Tiziano Caruso, Davide Pacifico

**Affiliations:** 1 Institute of Biosciences and Bioresources (IBBR), CNR, Via Ugo La Malfa 153, Palermo, 90146 Italy; 2 Department of Agricultural, Food and Forest Sciences (SAAF), University of study of Palermo, Viale delle Scienze, Palermo, 90128 Italy

**Keywords:** Olive tree, Climate change, Endophytes, Phyllosphere microbiota, Biological control, Microbial diversity, Sustainable agriculture

## Abstract

The olive tree (*Olea europaea* L.) hosts diverse endophytic microbial communities that contribute to its resilience, productivity, and adaptation to environmental stressors. Since the temperature increases caused by global climate change primarily affects the aerial part of the plant, this review synthesizes current knowledge on the diversity, composition, and ecological drivers of olive phyllosphere endophytes, with a focus on bacterial and fungal communities. We highlight the role of host-related factors—including plant genotype, organ specificity, age, and phenological stage—in shaping microbiota structure across spatial and temporal scales. Genotype consistently emerges as a major determinant of microbial composition, while leaves and twigs harbor distinct yet overlapping communities. Geographic location, environmental variables, and seasonal shifts significantly influence microbial assemblages, with closer sites often supporting more similar communities. We also discuss the impact of agricultural practices and biotic and abiotic stressors on microbiota stability and function. Notably, several cultivable taxa—including *Bacillus*, *Paenibacillus*, *Pantoea*, *Aureobasidium*, and *Penicillium*—exhibit antagonistic activity against key olive pathogens, underscoring their potential as biological control agents. We conclude by emphasizing the need for functional studies to elucidate the roles of keystone endophytes and to inform microbiome-based strategies for sustainable olive cultivation.

**Graphical Abstract:**

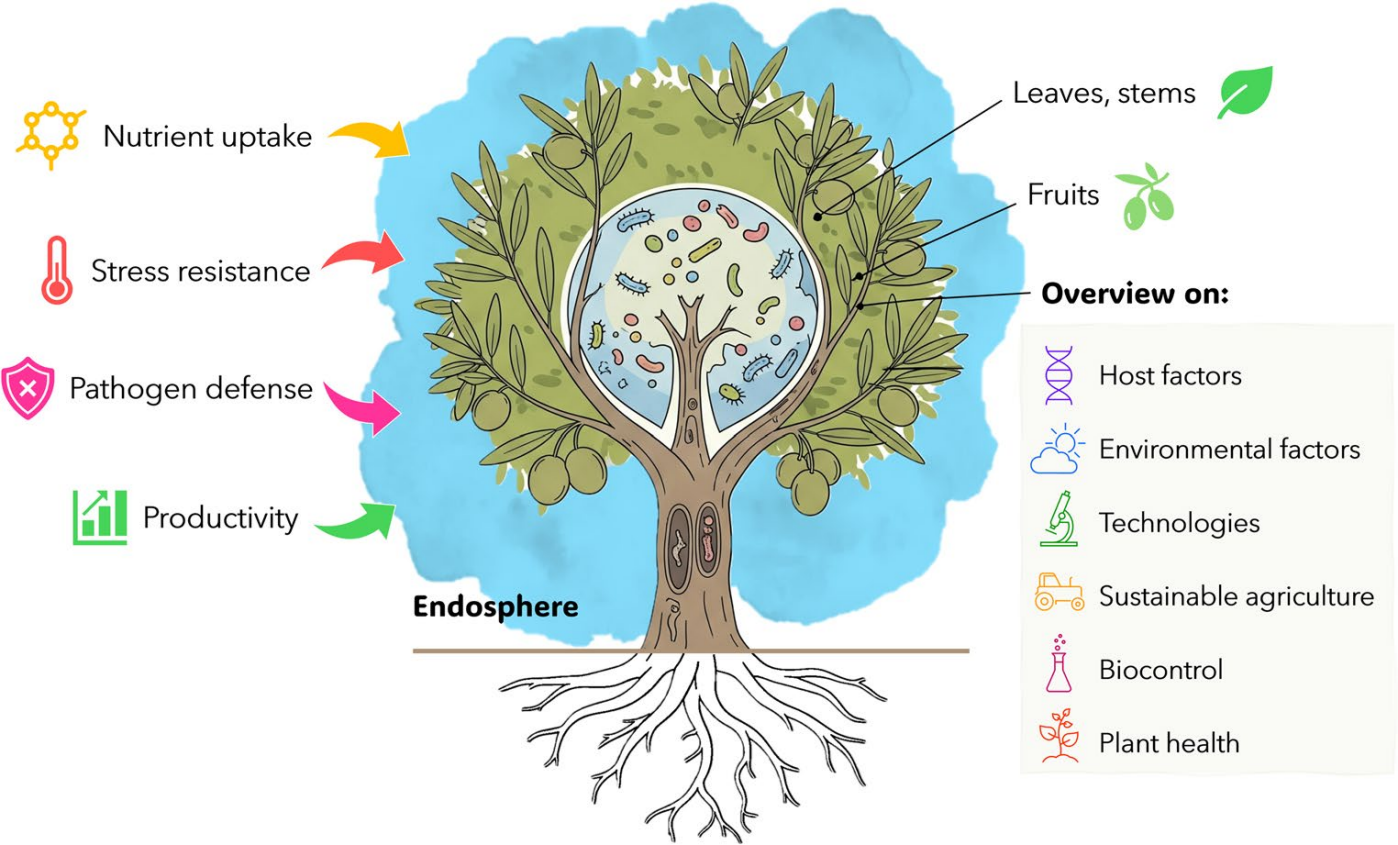

## Introduction

The olive tree (*Olea europaea* L.) has been cultivated for millennia across the Mediterranean basin, where it holds a central role not only in agriculture, but also in the economic and cultural heritage of the region [1].

Archaeological findings indicate that olive cultivation originated between the Southern Caucasus and the Near East around 6,000 years ago [2], and later spread throughout the Mediterranean by ancient civilizations [1]. Today, olive trees are grown across nearly 60 countries on five continents [3] and more than 2000 cultivars are known worldwide [4].

The genus *Olea* L. comprises 33 species of evergreen shrubs and trees naturally distributed throughout warm-temperate regions worldwide. Among them, *Olea europaea* L. is the only species that has been domesticated. Within this species, the subspecies *Olea europaea* subsp. *europaea* includes both the wild form (*var. sylvestris*) and the cultivated form (*var. europaea*) [3].

In recent decades, olive cultivation has progressively expanded into (semi)arid regions, including countries such as Turkey, Syria, and Saudi Arabia [3], highlighting the remarkable adaptability of this crop to diverse environmental conditions.

Renowned for its adaptability, the olive tree thrives under a wide range of environmental conditions, including those characterized by abiotic stresses such as prolonged drought [5], high soil salinity [6, 7], and nutrient-poor substrates [8]. Moreover, the global expansion of olive cultivation into new regions, together with the changing dynamics of plant-associated fauna driven by climate change, has increased the exposure of olive trees to emerging pests and diseases [3, 9]. To cope with this challenging environment, olive trees have evolved intricate relationships with their associated microbiota, particularly with the microbial communities inhabiting the phyllosphere [3].

The phyllosphere, the aerial habitat encompassing leaves, stems, flowers, and fruits, constitutes a dynamic ecological niche where plant tissues interface directly with surrounding microbial communities. This compartment supports a complex web of interactions, many of which are mediated by endophytic microorganisms that colonize internal plant tissues without eliciting disease symptoms. These endophytes contribute significantly to plant fitness by enhancing nutrient acquisition, conferring tolerance to abiotic stresses, and suppressing pathogens [10]. Unlike root-associated microbiota, which are predominantly shaped by edaphic properties such as soil composition and nutrient availability [11, 12], phyllosphere communities are primarily shaped by host genotype, environmental exposure, and seasonality [13].

Deciphering the composition and function of the olive phyllosphere microbiota is essential for harnessing its full potential in sustainable crop production. The increasing global interest in microbiome-informed strategies for enhancing plant resilience and productivity highlights the need for a deeper understanding of these microbial partners and their ecological roles. Recent developments in high-throughput sequencing and omics-based technologies have provided unprecedented insights into the taxonomic and functional diversity of bacterial and fungal communities in the olive phyllosphere [14–19], revealing their potential in promoting plant health and mitigating disease impact [20].

In this review, we focus specifically on the phyllosphere microbiota of olive trees, while fully recognizing the critical importance of root-associated microbial communities. Our intent is not to diminish the role of belowground microbiota, but rather to underscore the unique ecological features and functional roles of microbial communities inhabiting the aerial plant environment which are gaining increasing interest because they are more sensitive to climate change. Although the rhizosphere and phyllosphere are interconnected via plant physiological processes, they are shaped by distinct environmental filters and selective pressures, resulting in functionally and compositionally divergent microbiomes.

Root-associated endophytes are strongly influenced by soil texture, organic matter content, and microbial interactions in the rhizosphere [21], whereas phyllosphere microbes are primarily affected by environmental exposure, atmospheric dynamics, UV radiation, humidity, and temperature fluctuations, as well as host-derived factors such as surface exudates and cuticle characteristics [13]. Given these fundamental differences, we argue that a dedicated analysis of phyllosphere microbiota is necessary to accurately assess their contributions to olive tree health. Future research should aim to integrate both below- and above-ground microbial compartments in order to achieve a comprehensive understanding of olive-microbiome interactions. However, within the scope of this review, we offer a detailed examination of the phyllosphere endophytic microbiota and its ecological relevance, with a particular emphasis on its potential application in biological control and climate-resilient olive cultivation.

## Drivers of Olive Microbiota Communities

**Fig. 1:**
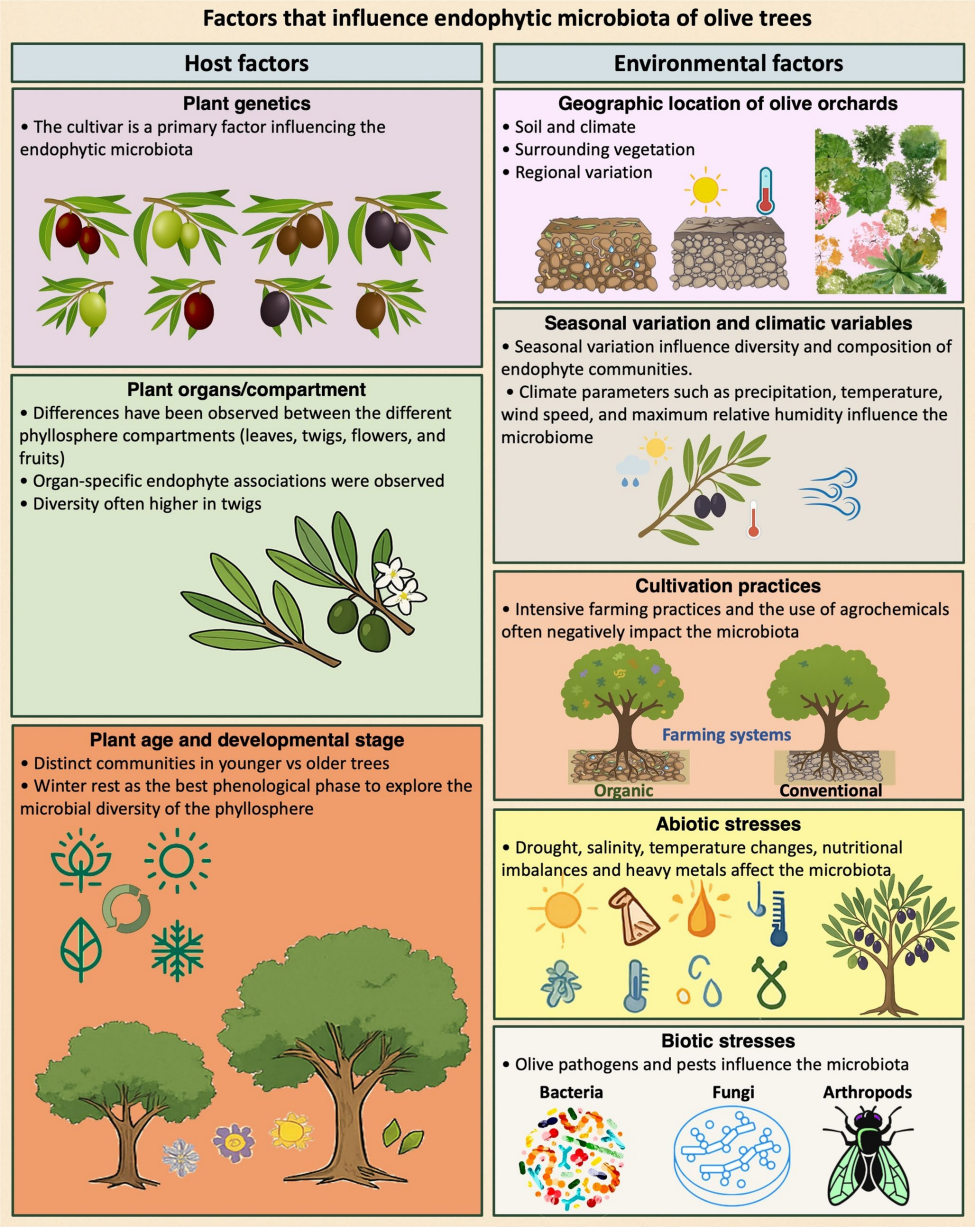
Host and environmental factors that influence the composition and diversity of the endophytic microbiota in the olive phyllosphere

The structure of the olive-associated microbiota results from intricate interactions among the host plant, microbial communities and a range of environmental drivers, including climatic conditions, agronomic practices, and both biotic and abiotic stressors. This section provides an overview of current scientific understanding concerning the main host and environmental factors that influence the composition and diversity of the endophytic microbiota in the olive phyllosphere (Fig. 1). The synthesis includes evidence from both classical plant–microbe interaction studies based on culturable microorganisms, and more recent meta-omics approaches, which have significantly expanded our capacity to characterize microbial diversity, structure, and function under varying ecological and genetic contexts.

### Host Factors Influencing Endophytic Microbiota of Olive Trees

#### Plant Genetics

Studies on the olive phyllosphere microbiota have been primarily conducted in Mediterranean countries with a well-established history of olive cultivation (such as Spain, Italy, Portugal, and Greece) and more recently in Brazil. These investigations, often adopting comparable sampling strategies and analytical pipelines, have produced a comprehensive depiction of the microbial assemblages associated with different olive genotypes. Olive orchards are recognized as rich microbial reservoirs, hosting a variety of bacterial and fungal communities across different cultivars and plant genotypes. According to several studies [14–19, 22–29], the olive phyllo-endosphere is predominantly inhabited by bacterial phyla such as Proteobacteria, Actinobacteria and Firmicutes, and fungal communities mainly from the phylum Ascomycota.

The role of host genotype and geographic origin in shaping the structure and function of endophytic communities was first highlighted by Müller et al. [15] through a study conducted in a Spanish olive orchard. The authors analyzed microbial communities from ten cultivated olive varieties of Mediterranean origin and nine wild olive accessions from Cyprus, Greece, and Madeira Island. Using 16 S rRNA gene amplicon sequencing, the study demonstrated a strong correlation between the microbial composition and the geographical origin of the olive genotypes, which reflected a broader differentiation between “Eastern” and “Western” Mediterranean regions. Notably, wild olive trees from the same regions as their cultivated counterparts harbored similar endophytic communities, suggesting the presence of a regional microbial signature conserved across domestication boundaries. The bacterial taxa were dominated by Alpha-, Beta-, and Gammaproteobacteria, followed by Firmicutes, Actinobacteria, and Bacteroidetes. Bacteria *Pelomonas sp.*, *Ralstonia sp.*, *Pseudomonas sp.*, and *Actinobacter sp.* were considered the main representatives of the putative core microbiota. Interestingly, the authors observed that the leaf tissues of all genotypes were highly colonized by endophytic Archaea, with the phyla Thaumarchaeota (class Thaumarchaeota) and Crenarchaeota (class MBG group A) being dominant, while Euryarchaeota (classes Methanomicrobia and Halobacteria) were less abundant. The thaumarchaeal genus *Nitrososphaera* contributed to the core microbiota, which was characterized by a high proportion of archaeal 16 S rRNA genes relative to the total prokaryotic 16 S rRNA gene copies. Moreover, the archaeal order Crenarchaeales was detected exclusively in olive genotypes originating from western Mediterranean regions—most notably in the Spanish cultivar ‘Ocal’—followed by Nitrososphaerales and Crenarchaeota. In line with metagenomic studies on roots and twigs of olive cultivars [30, 31] and on the internal tissues of herbaceous and shrubby plants [32], these findings suggest the existence of plant–Archaea interactions, with certain archaeal groups possibly being selected by olive trees and/or adapted to endospheric habitats. Plant-associated Archaea have never been cultured, and although molecular detection approaches have improved their taxonomic classification, further studies are needed to elucidate their functional role in plant development.

In Portugal, Mina et al. [25] examined the impact of plant genotype at cultivar level on bacterial communities within the phyllosphere. The study, based on culture-dependent techniques, was carried out in three orchards located in the Mirandela region, comparing two widely cultivated olive varieties: ‘Cobrançosa’ and ‘Verdeal Transmontana’. Results revealed that host genotype significantly influenced both the diversity and composition of bacterial communities. Specifically, ‘Verdeal Transmontana’ hosted a greater abundance and richness of endophytes, along with higher alpha diversity, compared to ‘Cobrançosa’. Distinct microbial signatures were associated with each cultivar: *Pseudomonas aeruginosa*, *Pseudomonas graminis*, and *Brevundimonas sp.* were more frequently recovered from ‘Cobrançosa’, whereas *Pantoea vagans*, *Pantoea brenneri*, and other *Pseudomonas* species predominated in ‘Verdeal Transmontana’. These findings support the hypothesis that host genotype acts as a selective filter in shaping the phyllosphere microbiome.

Anguita-Maeso et al. [26] analyzed the xylem sap microbiota of two widely cultivated Spanish olive varieties, ‘Arbequina’ and ‘Picual’, and reported significant differences in bacterial composition. Notably, a higher number of unique bacterial genera were detected in ‘Picual’, reinforcing the hypothesis that plant genotype exerts a selective influence on the structure of endosphere microbiota.

To further investigate genotype-driven variation across different plant compartments, Malacrinò et al. [18] examined bacterial communities in fruits, leaves and surrounding soil of two olive genotypes (cv. ‘Sinopolese’ and cv. ‘Ottobratica’), revealing that plant genotype was a key determinant in shaping microbiota composition. Each compartment harbored distinct microbial assemblages, and a significant genotype × compartment interaction was observed. The influence of genotype was particularly pronounced in fruit tissues, with *Pseudomonas* and *Escherichia-Shigella* more abundant in ‘Sinopolese’, and *Raoultella* and *Klebsiella* in ‘Ottobratica’.

In one of the earliest studies on fungal endophytes of olive fruits, Preto et al. [33] investigated two Portuguese cultivars with differing susceptibilities to olive anthracnose: the highly susceptible ‘Madural’ and the moderately tolerant ‘Verdeal Transmontana’. The fungal community composition and abundance varied significantly between cultivars. Cultivar ‘Madural’ had lower colonization rates and species evenness, with *Gibberella* dominating. In contrast, *Neofabraea vagabunda* was exclusive to ‘Verdeal Transmontana’, suggesting a genotype-driven selection of endophytic fungal communities. Further evidence for genotype effects on fungal diversity was provided by Materatski et al. [24], who isolated endophytes from olive leaves of three cultivars (‘Galega vulgar’, ‘Cobrançosa’, and ‘Azeiteira’) collected from different sites in the Alentejo region of southern Portugal. The results showed that both endophytic richness and diversity varied significantly by cultivar and location, with ‘Galega vulgar’ exhibiting particularly low evenness, indicating that host genotype interacts with local environmental conditions to shape fungal community structure.

Costa et al. [16] employed ITS1 amplicon sequencing to analyze the fungal communities of the phyllosphere (leaves and twigs) in five olive cultivars (‘Cobrançosa’, ‘Galega vulgar’, ‘Madural’, ‘Picual’, and ‘Verdeal Transmontana’). Their results confirmed that host cultivar was the most significant driver of fungal community assembly. Although taxa from the phyla *Ascomycota* and class *Dothideomycetes* were predominant across all cultivars, distinct fungal profiles emerged for each genotype, suggesting the coexistence of a conserved core microbiota and cultivar-specific components.

In a biogeographically distinct context, Ngubane et al. [34] conducted the first comparative study of fungal endophytes in two subspecies of *Olea europaea* from South Africa, namely the native African olive (*O. europaea* subsp. *cuspidata*) and the introduced cultivated European olive (*O. europaea* subsp. *europaea*). Twigs were sampled across six locations in the Western Cape Province. Native trees harbored more diverse and species-rich fungal communities. Moreover, distinct clustering patterns were observed between the two subspecies, with European cultivars forming more homogeneous groups. These findings highlight that host identity shapes endophytic assemblages and that cultivated varieties may lose or acquire endophytes during geographical relocation.

A genotype effect was also documented by Hanani et al. [28] in Apulia, southern Italy. The authors investigated culturable endophytes in the sapwood of three cultivars with varying susceptibility to *Xylella fastidiosa*: the resistant ‘Leccino’ and the susceptible ‘Ogliarola salentina’ and ‘Oliva rossa’. ‘Leccino’ exhibited significantly higher bacterial richness and a distinct profile of dominant genera such as *Bacillus*, *Methylobacterium*, and *Paenibacillus.* Fungal colonization and endophyte density also varied among cultivars. ‘Leccino’ hosted larger populations of commonly found genera including *Aspergillus*, *Cladosporium*, *Fusarium*, and *Pithomyces chartarum*. In contrast, de Oliveira et al. [17] found that the phyllosphere microbiota of five Brazilian olive cultivars (‘Arbequina’, ‘Arbosana’, ‘Ascolana’, ‘Koroneiki’, and ‘Grappolo’) was not significantly influenced by genotype in terms of abundance or composition. The authors attributed this to the wide geographic distances betw een sampling sites, which were located in different Brazilian states. Supporting the role of regional specificity, Crucitti et al. [19] employed high-throughput sequencing to assess endophyte diversity in twigs from three Sicilian cultivars (‘Nocellara del Belice’, ‘Nocellara Etnea’, ‘Nocellara Messinese’) as well as from wild olive trees (*O. europaea* var. *sylvestris*). While alpha diversity was not significantly influenced by host type, fungal community composition clearly diverged between wild and cultivated olives. Wild olives clustered separately and hosted unique taxa such as *Robbsia*, *Kineosporia*, *Bryocella*, *Phallus*, *Orbilia*, *Neopyrenopeziza*, *Bellamyces*, and *Stagonospora*. In a related culture-dependent study, Crucitti et al. [29] confirmed these findings: wild olives exhibited higher bacterial and fungal diversity than cultivated ones. Genera such as *Bacillus*, *Staphylococcus*, and *Quambalaria* were identified as part of the core culturable microbiota of Sicilian olives.

Overall, olive cultivar plays a pivotal role in structuring both fungal and bacterial endophytic communities in the phyllosphere. Some varieties tend to harbor richer and more diverse microbiota, others exhibit simpler communities dominated by a few taxa and others to select for more specific microbial communities, suggesting a genotype-specific effect in the recruitment and modulation of microorganisms. For example, some cultivars exhibit a greater relative abundance of certain bacterial taxa belonging to the genera *Pseudomonas* or *Bacillus*, known for their growth-promoting properties or potential role in biotic stress resistance. Other cultivars appear to host fungal communities with a greater presence of endophytes belonging to the genera *Cladosporium* or *Alternaria*, whose role in plant physiology can range from mutualistic to potentially pathogenic, depending on the ecological context. These patterns suggest that cultivar influences not only which microorganisms are able to colonize the phyllosphere, but also how these microorganisms interact with the host and with each other. These findings present interesting prospects for using cultivars as a tool to guide microbiome composition, with potential applications in sustainable agriculture and variety breeding.

The assembly of microbial communities associated with plants can be driven by host genetic traits and environmental factors, which together shape distinct plant phenotypes [35]. Microbiota biodiversity has been positively correlated with host species characteristics such as wood density, leaf nitrogen content, anatomical and physiological leaf traits, and the presence of specific secondary metabolites [36, 37]. This relationship has been confirmed even when environmental variability was controlled through common garden experiments [35].

Plant genetics is known to strongly influence the composition of root exudates, thereby shaping soil microbial diversity and community structure, and determining the selective recruitment of microorganisms in the rhizosphere [38]. Metabolomic and ionomic analyses of olive trees have further confirmed the effect of plant genotype and age on the variation of xylem-associated microbial communities [26]. For instance, concentrations of aspartic acid, phenylalanine, sodium, and iron in olive xylem sap differed among cultivars [26], as did the proline, carotenoid, and pigment contents in fresh leaves [39]. Although systematic evidence of the genotype’s influence on the overall plant metabolome and ionome remains limited, its role in modulating plant adaptation to various stresses through the regulation of metabolite homeostasis cannot be excluded.

Genetic variability among olive cultivars, as revealed by nuclear simple sequence repeat markers, has been shown to cluster cultivars into distinct genetic pools, explaining the observed variation in seed-associated bacterial communities [40]. Specific plant loci also contribute to shaping the plant microbiome through their functional gene expression: diverse gene pathways related to plant development, immunity, and nutrient uptake and transport can be exploited by plants to either promote or restrict microbial colonization [41]. Moreover, artificial selection for breeding traits may have disrupted the long-term coevolution between plants and their microbiota, leading to an altered microbial composition in modern crops compared to their wild relatives, which often display greater resilience and reduced susceptibility to stress due to their symbiotic associations in natural environments [42].

These differences may reflect variations in cultivar-specific features—such as tissue physics-chemistry, morphological traits, metabolite production, innate immune response and defense mechanisms—act as selective filters, shaping the composition and ecological function of resident microbial assemblages.

#### Plant Organs/Compartments

An important dimension in understanding endophytic diversity within the olive phyllosphere is the specific plant organ or tissue from which microbial communities are recovered. Different organs provide distinct microenvironments, potentially selecting for organ-specific microbial assemblages.

Phyllosphere is a dynamic habitat, and microbial-associated colonizers are subjected to varying conditions like temperature, nutrient and water availability, moisture, pH, and UV radiation which shape microbial communities. Also, these environmental elements have a direct effect on photosynthesis, plant respiration and hormonal profiles and indirectly affect the composition of microbiota [43]. Each organ, including roots, stems, leaves, flowers, fruits, and seeds, has unique biotic and abiotic factors that favor the selection of specific microorganisms with the right metabolic and resistance traits. For example, oxalate utilization, the production of quorum-sensing compounds, and nitrogen-fixing capabilities are some of the functional profiles required for the recruitment of beneficial endophytes from the rhizosphere, while the secretion of cell wall cellulolytic enzymes, the production of reactive oxygen species, and a cluster of genes involved in biofilm production, adhesion, motility, and chemotaxis become essential characteristics for tissue entry, plant colonization, and for leading an endophytic lifestyle within the host plant [44].

Mina et al. [25] investigated the influence of leaf versus twig tissues on culturable bacterial communities in the Portuguese cultivars ‘Cobrançosa’ and ‘Verdeal Transmontana’. They found that the effect of plant organ on bacterial composition was less pronounced than that of host genotype or the structure of the epiphytic microbiota. Nonetheless, some degree of organ-level differentiation was evident, with differences more evident in leaves than in twigs: *Ochrobactrum* was predominantly associated with leaf tissues, while *Brevundimonas* characterized twig-associated communities. The limited distinction between leaf and twig endospheres may be due to their shared physiological roles and similar endospheric conditions. Using a metabarcoding approach, Abdelfattah et al. [14] found that the endophytic fungal consortia associated with leaves, flowers, and fruits of *Olea europaea* were most distinct in the leaf samples, which clearly segregated from all other organs. The higher fungal colonization in leaves may be attributed to their constant presence on the tree, their large surface area relative to volume compared to fruits, and their longer lifespan compared to both flowers and fruits. Martins et al. [22], in a study across nine olive groves of cv. ‘Cobrançosa’ in the Bragança District (Northeast Portugal), and Costa et al. [16], analyzing several cultivars (‘Cobrançosa’, ‘Galega vulgar’, ‘Madural’, ‘Picual’, and ‘Verdeal Transmontana’) in the Iberian Peninsula, found that fungal colonization rates were higher in leaves than in twigs. However, the number of fungal taxa was greater in twigs. Although fungal communities from leaves and twigs formed a tightly clustered and compositionally similar group, some taxa were exclusively isolated from one organ: *Trichoderma gamsii*, *Trichoderma* sp. 1, *Epicoccum nigrum*, and *Penicillium canescens* were found only in twigs, while *Paraphoma chrysanthemicola*, *Alternaria arborescens*, *Alternaria alternata*, and *Penicillium restrictum* were exclusive to leaves. One possible explanation for the observed overlap between leaf and twig microbiota is the vegetative propagation of olive trees and their common exposure to aerial inocula, facilitating horizontal transmission of microbial taxa across organs. Despite this overlap, the structural and biochemical differences between tissues can still shape organ-specific microbial consortia. Several structural and biochemical leaf traits, such as leaf mass per area, thickness, cuticle properties, and carbon–nitrogen balance, as well as the content of secondary metabolites, can modulate microbe–host interactions, tissue colonization, and ultimately influence the diversity and composition of the endophytic community [45]. In olive trees, the leathery leaves are further enriched in triterpenes, mainly oleanolic and maslinic acids, which form a physical and chemical barrier, preventing microbial entry [46, 47]. Woody stems may provide specific substrates, such as cellulose, hemicellulose, and other structural carbohydrates, as well as more available nutrients, which can selectively favor endophytes, promoting their colonization and species richness [48]. Xylem represents a particularly selective niche, characterized by nutrient scarcity, fluctuating negative pressure, low oxygen levels, and the presence of numerous plant metabolites involved in resistance or tolerance to biotic and abiotic stresses [49–51]. For these reasons, to colonize internal plant tissues or exploit the open conduits of xylem vessels [44, 49], endophytes need to be able to implement various mechanisms, including the production of cell wall-degrading enzymes (CWDEs), lipopolysaccharides, and motility strategies such as chemotaxis and twitching.

Gomes et al. [23] also reported high compositional similarity between leaf and twig fungal endophytes in Portuguese olives but found significantly greater abundance, richness, and diversity in twig samples. This was hypothesized to result from the structural robustness of twig tissues, which may offer a more stable and protected niche, less affected by external environmental stressors compared to the more delicate and exposed leaf surfaces. A survey of approximately 600 published studies across different plant biomes revealed that both fungi and bacteria are more prevalent in the stems of woody plants than in other tissues [52]. Stems and bark are continuously exposed to various microbial inocula, carried by air, water, and dust, throughout the plant’s lifetime, thereby encountering a broader range of microbial propagules. In addition, dead bark tissues often remain attached to the plant for extended periods, further contributing to microbial colonization. In contrast, leaves typically remain on the plant for a shorter time, even in evergreen species. Moreover, older leaves tend to host more established endophytic communities than younger ones, likely due to their longer exposure to microbial inocula and the extended period available for microbial growth within the leaf tissues [53].

The greater endophytic richness associated with twigs was further confirmed by Crucitti et al. [29], who evaluated culturable endophytes in cultivated and wild Sicilian olive trees. The study showed that 35.7% of bacterial genera, including *Acinetobacter*, *Frondihabitans*, *Kocuria*, *Priestia*, and *Providencia*, and 55.5% of fungal genera, including *Alternaria*, *Acremonium*, *Chaetomium*, *Diaporthe*, *Didymella*, *Elsinoe*, *Geomyces*, *Nemania*, *Neosetophoma*, *Paraconiothyrium*, *Peniophora*, *Peziza*, *Phoma*, *Stemphylium*, and *Tricharina*, were isolated exclusively from twig tissues. These findings underscore the importance of twigs as a reservoir of endophytic diversity in the olive phyllosphere. Woody tissues, rich in structural carbohydrates, offer complex and selective carbon sources that can be exploited by specialized fungal and bacterial taxa. Furthermore, the internal environment of woody organs is relatively protected from external stresses enhancing microbial survival compared to the more exposed aerial surfaces. Dead or senescent bark layers may also act as transitional niches, supporting both epiphytic and endophytic microorganisms and facilitating microbial exchange between external and internal compartments. In contrast, leaves host more transient endophytic assemblages shaped by environmental variability, tissue age, and plant phenology, while root-associated microbiota is strongly influenced by soil conditions and rhizodeposition patterns. Therefore, branches can be considered long-term microbial reservoirs that integrate inocula over time and from multiple environmental sources (air, rain, arthropods, and adjacent tissues) that may contribute to recolonization of newly formed tissues and to the systemic protection and adaptability of the host plant.

Beyond vegetative tissues, reproductive organs have also been shown to harbor unique microbial assemblages. Martins et al. [27] analyzed flower buds, flowers, and fruits of cv. ‘Madural’ and found clear differences in fungal community composition and richness across developmental stages and tissue types. *Biscogniauxia mediterranea* and *Cladosporium cladosporioides* were predominant in flower buds, while flowers were characterized by *Pezizomycetes* sp. and *Diaporthe rudis*. Fruits hosted a distinct community, dominated by *Epicoccum nigrum*, *Trametes sp.*, and *Neofabraea vagabunda*. These results reinforce the idea that plant organs impose selective pressures that shape the composition and ecological roles of associated endophytic microbiota.

Wentzien et al. [40] investigated the composition of the olive seed microbiota in eight olive cultivars and two wild genotypes grown in the same orchard using Illumina sequencing. The olive seed endosphere harbored previously unexplored bacterial, fungal, and archaeal communities, whose structure and composition were influenced by the plant genotype. *Actinobacteria*, *Basidiomycota*, and *Ascomycota* were identified as the most abundant phyla, while the core microbiome consisted of four bacterial genera (*Stenotrophomonas*, *Streptomyces*, *Promicromonospora*, and *Acidipropionibacterium*) and three fungal genera (*Malassezia*, *Cladosporium*, and *Mycosphaerella*). Among them, the bacterial genus *Streptomyces* and the fungal genus *Malassezia* emerged as distinctive signatures of the olive seed microbiota. Although the role of seed endophytes remains to be fully elucidated, such vertical transmission ensures the inheritance of putative beneficial endophytes by young seedlings, where they can contribute to breaking seed dormancy, promoting germination and growth, and protecting the young plant from biotic and abiotic stresses [54, 55].

#### Plant Age and Developmental Stage

The influence of olive tree age on the composition and diversity of associated microbial communities has been, to date, only marginally investigated. One of the first studies addressing this factor was conducted by Anguita-Maeso et al. [26], who compared the xylem sap microbiota of adult olive trees (10 years old) and young plantlets (1 year old) of the cultivars ‘Picual’ and ‘Arbequina’. Their results revealed significant age-related differences in microbial assemblages. Specifically, adult trees exhibited higher bacterial diversity, as measured by Shannon alpha-diversity, and a greater number of unique bacterial genera not shared with their younger counterparts. To further evaluate the influence of age, de Oliveira et al. [17] analyzed microbial diversity and composition in Brazilian olive orchards. Leaf-associated bacterial communities from eight-year-old trees differed significantly from those of four-, five-, and seven-year-old trees. Both alpha and beta diversity of associated fungal communities also varied with plant age. The main microbial taxa contributing to these differences were *Stenotrophomonas* and *Achromobacter* (bacteria), and *Pseudocercospora*, *Hyphozyma*, and *Symmetrospora* (fungi). These findings underscore the importance of further research on age-related microbiota shifts in olive trees, ideally involving a broader range of genotypes and agronomic conditions.

These findings suggest that microbial community structure evolves over time, likely influenced by cumulative environmental exposures and physiological changes in the host. Age-related differences in phyllosphere endophytes may be attributed to changes in leaf and cuticle structure, trichome distribution, volatile compound profiles, hormone levels, and other age-dependent physiological factors.

Microbial community dynamics have also been explored across different developmental (phenological) stages of the olive tree. Crucitti et al. [19] analyzed alpha diversity in three Sicilian cultivars (‘Nocellara del Belice’, ‘Nocellara Etnea’, and ‘Nocellara Messinese’) and wild olives during winter dormancy, full bloom, fruit set, and fruit ripening. Bacterial species diversity and evenness varied significantly across growth stages, with the highest values recorded during winter dormancy. In a complementary culture-dependent study, Crucitti et al. [29] isolated bacteria and fungi from leaves and twigs of the same cultivars and wild genotypes, confirming winter as the most productive season in terms of microbial genera recovery, with a predominance of fungal taxa such as *Pyronema*.

The influence of the development stage of the olive host on fungal microbiota has been previously investigated. In the first metabarcoding study on this topic, Abdelfattah et al. [14] monitored the fungal diversity of the phyllosphere and carposphere of cv. ‘Ottobratica’ at four phenological stages (May, June, October, and December). In the phyllosphere, dominance values increased progressively from May to December, while Shannon diversity showed notable variation in flower samples during May, likely due to the transition from flower to fruit. *Devriesia* and *Pseudocercospora* were dominant in October and December, while *Aureobasidium* and *Cladosporium* were more prevalent during June.

The dynamics and the underlying processes regulating the endophyte variation in olive trees remain largely unexplored, highlighting the need for further in-depth and geographically diverse investigations.

### Environmental Factors as Drivers of Endophytic Microbiota Assembly of Olive Tree Phyllosphere

Geographic location and local environmental conditions are key determinants of endophytic microbiota structure and diversity in olive trees. These factors often exert a greater influence than topographic variables such as altitude, particularly when sites differ in land use, vegetation cover, or microclimatic conditions.

#### Geographic Location of Olive Orchards

Endophytic bacteria associated with olive leaves in the southeastern region of South America were recently identified by de Oliveira et al. [17]. Their study revealed that the diversity of microbial communities in five different cultivars was affected more by geographic location than by altitude, specifically based on differences of position among the six and three farms in the states of São Paulo and Minas Gerais, respectively. Regarding microbial composition, counties could be distinguished based on the presence of *Stenotrophomonas* and *Achromobacter*. Similarly, both geographic location and altitude affected the diversity, abundance, and composition of fungal communities, with *Hyphozyma* and *Pseudocercospora* identified as the main contributors to inter-county differences for both factors.

Comparable patterns have been observed in Portugal. Martins et al. [22] assessed fungal endophyte diversity in leaves of cv. ‘Cobrançosa’ across three locations in the Trás-os-Montes region: Mirandela, Bragança, and Carrazeda de Ansiães. Fungal richness was highest in Mirandela, and community composition clustered distinctly by location. The distance between sites was positively correlated with dissimilarity in microbial profiles, with *Phomopsis columnaris* and *Fusarium oxysporum* identified as key taxa differentiating the three fungal communities. In southern Portugal, fungal endophyte diversity also varied considerably among olive cultivars across three sites: Vidigueira, Monforte, and Elvas [24]. The lowest fungal richness was recorded in Elvas, indicating a distinct endophyte profile compared to the other two locations and confirming high spatial variability in endophyte diversity.

At broader geographic scales, endophytic microbiota tends to display non-random regional distributions. Factors such as biogeography and soil physicochemical properties likely play important roles in shaping microbial communities, especially when production sites differ significantly in environmental conditions. In general, closely located sites with similar vegetation types often support more similar microbial communities. Ngubane et al. [34] found that fungal abundance and community composition in cultivated European olives and surrounding native African olives differed among South African sites, with less variation among nearby locations and those sharing similar vegetation. Since the environment is the primary source of many plant-associated endophytes, local factors such as geographic location and surrounding flora can have a major influence on endophyte community composition [56].

#### Seasonal Variation and Climatic Variables

The influence of seasonal changes on olive phyllosphere endophytes has been investigated. Seasonal variation in phyllosphere microbiota has been documented in a limited number of studies, with a focus on both culturable and unculturable fractions. Hanani et al. [28] assessed culturable endophytes in the sapwood of three cultivars sampled in fall (November), winter (February), and summer (July). They observed pronounced seasonal shifts in bacterial communities, with peak richness in summer. Interestingly, the microbial profiles from fall were more similar to those of winter, suggesting a possible dormancy or stabilization effect during colder months. Shifts in plant endophytic bacterial communities may be driven by the optimal growth temperatures of microbial species, by the developmental stage of the host plant, and by external temperature fluctuations [57, 58]. Variations in temperature, precipitation, solar radiation, and relative humidity can influence the activity of specific microbial taxa and simultaneously alter the physiological state and stress levels of the host plant. Such environmental changes modify the internal microenvironment within plant tissues, affecting the production and concentration of soluble sugars, proteins, amino acids, organic acids, other nutrients, and phytohormones. These biochemical and physiological shifts ultimately lead to seasonal fluctuations in bacterial richness and diversity, in the relative abundance of dominant genera, and in microbial co-occurrence patterns.

In Portugal, Martins et al. [22] studied seasonal variation in culturable fungal endophytes from cv. ‘Cobrançosa’ across several groves. Sampling occurred once per site from late spring (June) to autumn (November). Colonization frequency and abundance increased from June to November, while fungal richness and diversity decreased. Additionally, the community structure changed: in June, *Phomopsis columnaris* represented 47% of isolates, but dropped to 36% in November, while *Fusarium oxysporum* increased to 35%. *Trichoderma* sp. 1, absent in June, became the third most abundant isolate in November (20%).

In a study conducted in Mirandela [23], three olive orchards of different cultivars were sampled during two seasons: autumn (October–November) and spring (March–May). In line with earlier culture-based findings, fungal species richness, diversity (Simpson index), and community composition were significantly greater in spring than in autumn. Specific endophyte families were more strongly associated with spring (*Pyronemataceae*, *Pleosporaceae*, *Pezizaceae*) or autumn (*Leptosphaeriaceae*, *Trichocomaceae*).

In southern Portugal, Materatski et al. [24] examined seasonal variation in fungal endophytes of three cultivars (‘Galega vulgar’, ‘Cobrançosa’, and ‘Azeiteira’) sampled during spring, summer, and autumn. Unlike previous studies, they observed an increase in the number of isolated fungal operational taxonomic units (OTUs) from spring to autumn, with a significant rise in diversity and dominance in the latter season. These findings suggest that autumn had the strongest influence on fungal endophyte community structure and richness.

This discrepancy with earlier Portuguese studies (e.g., Martins et al. [22],; Gomes et al. [23]),, which found higher diversity in spring, may be attributed to differences in sample size and geographic location. Materatski et al. [24] sampled 270 trees over three seasons, compared to 63 trees in one-time-point sampling by Martins et al. [22], and two seasonal time points by Gomes et al. [23]. Furthermore, Materatski’s study was conducted in southern Portugal, a region with different climatic conditions—particularly in terms of rainfall and humidity—than the mountainous Bragança and Mirandela areas in the northeast. As reported in the literature, higher rainfall and relative humidity positively influence fungal endophyte colonization and dispersal.

These findings suggest that some fungal endophytes are progressively established over time, while others may decline or disappear, possibly due to interspecific competition or changes in plant tissue chemistry during phenological transitions. From summer to winter, olive trees undergo marked biochemical changes, including a decline in starch and soluble sugar content, the translocation of nitrogen to other tissues prior to leaf senescence, and an increase in the levels of certain amino acids (such as glutamate, aspartate, and γ-aminobutyrate) [59] and phytohormones, including auxins and cytokinins [60]. Amino acids promote fungal growth and metabolism, while phytohormones can facilitate fungal entry into plant tissues; carbohydrates, in turn, represent a primary carbon and energy source for these microorganisms [61–63]. Such seasonal fluctuations in plant metabolites may therefore account for the higher colonization rates and abundance of endophytic fungi observed during the winter months, albeit often accompanied by a reduction in fungal diversity.

Among the limited studies highlighting the role of climate in shaping microbial communities in detail, Gomes et al. [23] assessed the effects of various microclimatic parameters, including mean temperature, maximum and minimum relative humidity, cumulative rainfall, and mean wind speed, on culturable fungal assemblages in olive cultivars in Mirandela. The most influential variable was rainfall, followed by mean temperature, wind speed, and maximum relative humidity, all of which contributed to variation in the endophytic community. Among the climatic variables examined, rainfall best explained the variation in the overall structure of the fungal community. Rainfall contributes to shaping the endophytic mycobiota by promoting the dispersion of fungal spores and creating high-humidity conditions that favor fungal germination and growth. In addition, higher mean temperatures can enhance the viability of fungal propagules, thereby facilitating successful colonization of plant tissues [64, 65]. The combined influence of rainfall and temperature likely drives the seasonal turnover of endophytic fungi, selecting for taxa with physiological traits adapted to fluctuating moisture and thermal conditions, and ultimately shaping the functional composition of the fungal community within host tissues.

#### Cultivation Practices

The expansion of olive cultivation and the growing global demand for olives and olive oil have driven the adoption of high-density orchard systems to increase yield. Most olive groves are managed conventionally, often involving intensive use of agrochemicals and practices that negatively impact soil health and biodiversity.

Agricultural management practices influence the diversity, composition, and functionality of plant microbiota, exerting distinct effects on bacterial and fungal assemblages. Conventional management, characterized by intensive tillage, mineral fertilization, and pesticide application, often leads to a reduction in overall biodiversity [66]. Such practices tend to favor copiotrophic bacterial taxa that are tolerant to chemical and stress inputs, while diminishing endophytic populations associated with plant growth promotion and nitrogen fixation in the plant roots and aerial parts [67]. In contrast, organic and sustainable systems, which enhance soil biological activity and minimize synthetic inputs, support a more diverse and balanced endophytic bacterial community. These environments promote oligotrophic taxa capable of improving nutrient uptake, inducing systemic resistance, and producing bioactive compounds that contribute to host stress tolerance [67, 68].

Fungal endophytes appear to be even more sensitive to management-driven changes in organic matter of soil [67]. Organic management favors the colonization of endophytic fungi with saprotrophic or mutualistic functions [69, 70], conversely, intensive chemical treatments can suppress beneficial fungal guilds, leading to simplified communities dominated by opportunistic taxa [29, 71].

Few studies have investigated the impact of cultivation practices on the phyllosphere endophytic microbiota of olive trees, and further research is required to gain a more comprehensive understanding of the overall endophytic biodiversity. Microbial isolation and advanced molecular techniques, including next-generation sequencing, remain fundamental and complementary tools for elucidating microbial diversity. Through the isolation and characterization of microorganisms, it is possible to explore microbial diversity in relation to their ecological functions and potential biotechnological applications [72]. Moreover, culture-independent molecular approaches have revolutionized microbiome studies by revealing the hidden diversity of previously unculturable taxa [73].

Pascazio et al. [74] investigated the influence of farming systems on the carposphere bacterial endophytes of cv. ‘Maiatica’. Using 16 S rRNA fingerprinting by PCR-DGGE, they compared olive pulp (mesocarp) microbiota after 13 years of sustainable versus conventional management in Ferrandina (Basilicata, Italy). Their results showed that sustainable farming supported a greater number of bacterial species, with *Rahnella spp.*, *Kluyvera intermedia*, *Averyella dalhousiensis*, *Pantoea sp.*, and *Serratia sp.* (Enterobacteriaceae) being the most abundant.

Crucitti et al. [19], by Illumina next-generation sequencing, studied endophytic microbiota in the phyllosphere of Sicilian olive cultivars under organic and conventional farming systems. Bacterial species richness and phylogenetic diversity varied significantly between management types. In organic orchards, where *Wolbachia* and *Sulfitobacter* (Alphaproteobacteria) dominated, bacterial diversity was significantly lower than in conventionally managed orchards. In contrast, fungal community diversity showed no significant differences between the two systems.

Further analysis by Crucitti et al. [29] showed that the diversity of culturable endophytes also varied depending on cultivar and farming method. Organically farmed cv. ‘Nocellara del Belice’ and conventionally farmed cvs. ‘Nocellara Etnea’ and ‘Nocellara Messinese’ contributed most to overall endophytic isolate diversity, suggesting cultivar-specific responses to management practices.

Overall, the type of agricultural management not only influences the taxonomic composition of endophytic bacterial and fungal communities but also affects their ecological functions within the olive tree host. In particular, the organic farming system was characterized by a higher representation of sulfate-respiring bacteria and showed the greatest abundance of fungal taxa belonging to saprotrophic, pathotrophic, and symbiotrophic guilds [19, 29].

#### Abiotic Stresses

Environmental changes cause phenotypic shifts in plants, which in turn influence the structure of their associated microbiota. Endophytic microorganisms can support plant adaptation to various abiotic stresses, such as drought, salinity, temperature extremes, nutrient imbalances, and heavy metal contamination [75].

Using a metabarcoding approach, Vita et al. [39] documented a significant shift in the leaf endophytic bacterial community of olive trees exposed to moderate salt stress, but not under extreme salt stress conditions. Salt stress notably altered the abundance of bacterial endophytes in the four tested cultivars (‘Frantoio’, ‘Leccino’, ‘Oliana’, and ‘Lecciana’), with *Burkholderiales* and *Pseudomonadales* showing contrasting trends. ‘Oliana’ and ‘Lecciana’ under moderate salt stress also exhibited reduced community diversity. The osmotic changes induced by salinity likely selected for genera such as *Burkholderia* and *Ralstonia*, which are capable of thriving under such conditions.

Interestingly, while bacterial endophytes were negatively affected by high salinity, fungal communities in soil samples appeared to respond more positively, often replacing bacterial populations under extreme stress [76]. This contrasting response may reflect the greater tolerance of fungi to salinity compared with bacteria. The chitinous structure of fungal cell walls offers effective protection against water loss under low soil moisture and may also contribute to their enhanced resistance to osmotic stress caused by high solute concentrations [76]. Fungi may act as key contributors to ecosystem resilience and an in-depth investigation of fungal endophytic communities in olive trees would be valuable for elucidating their ecological and functional roles, as well as their potential interactions with the host plant under salt stress conditions.

To date, salinity is the only abiotic factor investigated for its effect on both the phyllosphere and carposphere endophytes in olive trees. More research is needed to assess the impact of other abiotic stresses and to fully understand the role of microbial communities as key allies for plant health and sustainable agriculture.

#### Biotic Stresses

Knowledge regarding the diversity and composition of olive-associated endophytes, and how microbial community shifts might select for keystone taxa in response to pathogen or pest invasion, remains incomplete. In this context, the first study to explore fungal communities in olives from cultivars with differing susceptibility to anthracnose, though asymptomatic, was conducted by Preto et al. [33]. Anthracnose, caused by several *Colletotrichum* species, primarily affects fruits, ranging from latent infections during flowering to a necrotrophic phase upon ripening [77]. Prior to fruit ripening, these pathogens may exist in the host as endophytes or hemi-biotrophs. In cv. ‘Madural’, the olive fruit endosphere contained *Colletotrichum* spp., confirming the cultivar’s susceptibility to anthracnose. Conversely, in the tolerant cv. ‘Verdeal Transmontana’, *Colletotrichum* was not isolated, though another phytopathogen, *Neofabraea vagabunda*, the causal agent of olive leprosy, was found in high abundance. This contributed to the differing microbial composition of endophytes among cultivars. Although no direct correlation was found between cultivar resistance and endophytic fungal community structure, each cultivar shaped its own distinct fungal microbiota, both in terms of phytopathogenic and total fungal communities.

Differences in fungal as well as bacterial endophytic communities among cultivars with varying levels of disease tolerance may involve competition for ecological niches and nutrients, as well as the production of antimicrobial compounds or signaling molecules that modulate community structure and host responses. Over time, such selective pressures may shape a distinct “microbial signature” characteristic of each cultivar, potentially contributing to its overall disease tolerance.

Similarly, the culturable endophytic fungal community of cv. ‘Madural’ was assessed in two orchards with contrasting levels of anthracnose incidence and severity [27]. Asymptomatic flower buds, flowers, and fruits were collected across the flowering-to-fruit set period. Fungal endophyte abundance and richness were significantly greater in the orchard with low anthracnose incidence, particularly in flowers (both abundance and richness) and fruits (abundance). In high-disease orchards, beneficial fungi were notably reduced in fruit tissues, while commensal fungi were significantly more abundant in flower buds and flowers of low-incidence orchards. Mycobiota composition also differed between orchards, with *Biscogniauxia*, *Cladosporium*, and *Colletotrichum* dominating in high-incidence orchards, whereas *Alternaria* and *Biscogniauxia* prevailed in low-incidence ones. Furthermore, the endophytes *Pseudophaeomoniella oleae*, *Neofabraea vagabunda*, and *Parastagonospora avenae* were positively correlated with *Colletotrichum* spp. in high-incidence sites. The decreased abundance of beneficial fungi may result from antagonistic interactions with the pathogen or be influenced by the presence of other plant pathogens.

Other major fungal pathogens affecting olive canopy include *Venturia oleaginea* (causal agent of peacock spot) and *Pseudocercospora cladosporioides* (responsible for cercosporiosis). Varanda et al. [78] used culture-dependent and -independent methods to investigate leaf mycobiota in three cultivars with differing susceptibilities to these diseases, sampling both symptomatic and asymptomatic trees. In southern Portugal (Alentejo region), the most widely grown cultivars (‘Galega vulgar’, ‘Cobrançosa’, and ‘Picual’) are considered resistant, moderately susceptible, and highly susceptible to *V. oleaginea*, respectively. Excluding the pathogens themselves, fungal richness generally declined in symptomatic trees of ‘Cobrançosa’ and ‘Picual’, whereas it increased significantly in ‘Galega vulgar’. The resistant cultivar exhibited significantly higher fungal richness and diversity, with five exclusive fungal genera (*Bullera*, *Cryptococcus*, *Fusicladium*, *Saccharata*, and *Sporobolomyces*), along with *Botrytis*. These taxa contributed to higher fungal richness in symptomatic trees of ‘Galega vulgar’, suggesting better adaptation and potential antagonistic activity in this cultivar.

Similar observations were made by Vergine et al. [79], who characterized the autochthonous fungal and bacterial endophytic microbiota of the resistant cv. ‘Leccino’ and the susceptible cv. ‘Cellina di Nardò’, both infected and uninfected with *Xylella fastidiosa*, a xylem-limited bacterium causing Olive Quick Decline Syndrome in southern Italy. Endophytic bacterial diversity in leaves was significantly higher in ‘Leccino’ than in ‘Cellina di Nardò’, while for fungal community the differences were less pronounced. The total endophytic microbiota of ‘Leccino’ remained stable regardless of infection, in contrast to ‘Cellina di Nardò’, where significant shifts occurred in infected trees. Infected ‘Leccino’ samples hosted bacterial taxa (*Silanimonas*, *Xanthomonadaceae*) absent in healthy samples. Two fungal genera, *Neodevriesia* and *Sphaceloma*, were negatively correlated with *X. fastidiosa*, suggesting potential inhibitory roles.

A comprehensive study by Giampetruzzi et al. [31] employed whole metagenome shotgun sequencing (WMSS) and metataxonomics to analyze xylem microbiomes in infected twigs of the susceptible cv. ‘Kalamata’ and resistant cv. ‘FS17’ during spring and autumn. While *X. fastidiosa* levels remained low in ‘FS17’, they increased sharply in ‘Kalamata’, comprising over 50% of total microbial reads in autumn. This increase in pathogen abundance was linearly correlated with overall bacterial population growth, suggesting pathogen dominance of the bacterial niche in susceptible cultivars. Fungal communities were shaped more by seasonal and environmental factors than by cultivar susceptibility or pathogen load. Overall microbial communities differed more in ‘FS17’ than in ‘Kalamata’, especially in spring and in plants with low *Xylella* levels, indicating that the pathogen influences microbiome dynamics more strongly in susceptible hosts. The authors took into account the archaeal microbiota represented mostly by the phylum Euryarchaeota (80.1%), followed by Crenarchaeota (11.2%) and Thaumarchaeota (8.7%). However, it was observed that archaeal communities were not driven by any of the factors considered (cultivar, season and pathogen).

Hanani et al. [28] corroborated the stability of sapwood endophytic communities in resistant cultivars by comparing culturable bacteria and fungi in one resistant (‘Leccino’) and two susceptible (‘Ogliarola salentina’,'Oliva rossa') cultivars. Bacterial richness declined significantly in infected ‘Ogliarola salentina’, whereas it remained stable in ‘Leccino’. Fungal endophyte dynamics were not influenced by cultivar susceptibility or symptom severity. In ‘Leccino’, *Curtobacterium*, *Bacillus*, *Pantoea* (bacteria), and *Paraconiothyrium*, *Pithomyces*, *Cladosporium* (fungi) were abundant regardless of health status, making them promising candidates for biocontrol against *Xylella fastidiosa*, a role already supported by studies in citrus and wheat [80, 81].

Vergine et al. [82] further analyzed the phyllosphere microbiota of genotypes with contrasting responses to *X. fastidiosa* in Salento. Asymptomatic or paucisymptomatic plants were compared to symptomatic ‘Cellina di Nardò’. Metabarcoding revealed that endophytic structure and diversity were shaped by the presence of *X. fastidiosa* subsp. *pauca*, especially among fungi. Asymptomatic genotypes hosted overrepresented fungal genera (*Pseudocercospora*, *Xenosonderhenioides*) and underrepresented ones (*Venturia*, *Fusicladium*, *Hormodochis*, *Paracucurbitaria*). Susceptible plants exhibited marked reductions in dominant xylem-associated bacterial genera (*Burkholderia*, *Dolichospermum*, *Bacillus*, *Hymenobacter*, *Sphingomonas*) and changes were also observed for several fungal taxa (such as *Lembosiella*,* Myriospora*,* Recurvomyces*,* Quambalaria*,* Phaffia*, and *Phaephleospora*). The persistent presence of beneficial endophytes such as *Burkholderia*, *Quambalaria*, *Phaffia*, and *Rhodotorula* in asymptomatic plants highlights the potential role of endophytes in disease tolerance.

Recently, Vergine et al. [83] demonstrated that infection by *Xylella fastidiosa* subsp. *pauca* (*Xfp*) modulates the endosphere microbiota of olive trees. The authors investigated the diversity and composition of endophytic communities in the canopy of both resistant (‘Leccino’) and susceptible (‘Cellina di Nardò’) olive cultivars, grouped according to different *Xfp* titers (control, low, and high). Proteobacteria and Ascomycota were the predominant groups in control plants, although microbial composition varied depending on the cultivar and infection level. At the genus level, *Pseudomonas*, *Acremonium*, and *Aureobasidium* dominated in healthy plants, regardless of cultivar. In infected plants, the bacterial microbiota was reshaped, with *Enterobacter* emerging as the dominant genus. The presence of the fungal genus *Acremonium* exhibited cultivar-dependent behavior: its abundance increased in the resistant cultivar and decreased in the susceptible one following *Xfp* infection. Similarly, *Aureobasidium* content increased in ‘Leccino’ in response to infection, while in ‘Cellina di Nardò’ its abundance varied with plant health status. Overall, microbial biodiversity was more uniform in the resistant cultivar, showing only minor fluctuations across health conditions. In contrast, high *Xfp* infection caused significant changes in the susceptible cultivar, increasing bacterial diversity while reducing fungal diversity. Moreover, ‘Leccino’ exhibited a higher abundance and diversity of beneficial endophytes, which helped mitigate some of the pathogen’s effects, highlighting their potential role in sustainable agriculture.

In plants, microbial diversity creates a robust, multi-layered defense, where diverse microorganisms act with different protective functions, being more resilient and effective than a single species [84]. High microbial diversity enhances microbial interaction and competition for space and nutrients within host tissues, limiting pathogen establishment and proliferation. In addition, diverse endophytic assemblages are more likely to include taxa that produce antimicrobial metabolites, lytic enzymes, or volatile organic compounds that directly inhibit pathogen growth [85]. Moreover, beneficial endophytes can modulate the host’s immune responses by priming defense pathways and improving stress tolerance, thereby strengthening systemic resistance against invading pathogens [85]. Collectively, these mechanisms create a multifaceted barrier that suppresses pathogen activity and contributes to plant health and disease resilience.

Olive knot disease (OKD), caused by *Pseudomonas savastanoi* pv. *savastanoi* (Psv), is another major bacterial threat in the Mediterranean region. Gomes et al. [86] examined fungal endophytes isolated from three cultivars with different OKD susceptibilities. Fungal diversity was highest in the moderately tolerant cv. ‘Cobrançosa’ and lowest in the highly susceptible cv. ‘Verdeal Transmontana’. The presence of knots (infected twigs) decreased fungal diversity in all cultivars, with the most notable drop in the tolerant cultivar. Host-pathogen interactions and cultivar-specific endophyte communities, likely shaped by resident microbiota, tissue chemistry, or competitive interactions, appear to influence fungal community structure.

Endophytic community composition can be markedly influenced by the biochemical and physiological alterations that occur in plants during pathogen infection. Changes in plant metabolite profiles may differentially favor or suppress specific microbial taxa. Opportunistic or saprophytic fungi tend to proliferate in response to elevated levels of soluble sugars and amino acids, whereas resistant microbial species are often selected under conditions of increased phenolics, alkaloids, or terpenoids [87]. During the priming phase of defense, only stress-tolerant or detoxifying endophytes are able to persist within the selective phytohormonal and oxidative environment [88]. Modulation of the host immune system further alters local nutrient fluxes and cell wall composition, thereby reshaping the ecological niches available for microbial colonization [89, 90]. Additionally, signaling molecules released by pathogens can interfere with endophytic responses and disrupt interspecies communication through quorum sensing [91].

Licciardello et al. [92] investigated whole endophytic communities in twigs of the highly susceptible cv. ‘Giarraffa’ and resistant cv. ‘Zaituna’ to OKD. Metataxonomic analyses showed higher bacterial richness but lower diversity in ‘Giarraffa’, and vice versa for fungi in ‘Zaituna’. Distinct endophytic profiles emerged, with *Amnibacterium*, *Methylobacterium*, *Sphingomonas*, and *Pithomyces chartarum* dominating in ‘Zaituna’, and *Alternaria*, *Neofusicoccum*, *Ascochyta*, *Elsinoë*, *Devriesia*, *Pseudocercospora*, and *Epicoccum nigrum* in ‘Giarraffa’. These findings suggest that host susceptibility influences pathobiome structure, likely through differential interactions between resident endophytes and the pathogen.

Although this review focuses on changes in phyllosphere microbial communities of infected olive trees, it is important to note that arthropods, nematodes, viruses, phytoplasmas, and oomycetes can also influence network complexity and the abundance of keystone taxa [3] Further studies are needed to elucidate the significance of shifts in endophytic community structure within the olive tree canopy for agricultural purposes.

Olive orchards are constantly threatened by major insect pests feeding on phyllosphere organs, such as the olive fruit fly *Bactrocera oleae* (Diptera, Tephritidae), the olive moth *Prays oleae* (Lepidoptera, Hyponomeutidae), and the olive black scale *Saissetia oleae* (Homoptera, Coccidae). A few metabarcoding-based studies have shown that microbiota associated with these olive insect pests share several bacterial and fungal taxa frequently detected in olive tissues, including *Bacillus*, sooty mold fungi, and plant pathogens [93, 94].

Only Abdelfattah et al. [95] evaluated the impact of *Bactrocera oleae* infestations on the fungal communities of infested and non-infested olive drupes using Illumina sequencing. The results revealed a general reduction in fungal diversity and an increase in unidentified or poorly characterized taxa, as well as in taxa that are usually outcompeted. Moreover, fly infestation disrupted the natural microbial balance, particularly affecting low-abundance taxa.

To date, fifteen viruses from nine genera have been identified as capable of infecting olive trees, mainly causing leaf yellowing and shoot deformation [3]. Although specific studies are lacking, it is reasonable to assume that viral infections may induce dysbiosis in the endophytic microbiota of olive trees, leading to imbalances in microbial diversity and altering the complex network of microbial interactions.

In Iran, Russian olive trees were reported to host co-infections of various phytoplasma species, with *‘Candidatus Phytoplasma asteris’* identified as the dominant one [96]. Next-generation sequencing analyses revealed a diverse and unique composition of both prokaryotic and eukaryotic species within infected plants. Among them, *Mycoplasmatota* was found to be the most abundant group, followed by endosymbiotic bacteria. Interestingly, viral genomes and archaeal (*Methanobacteria*) genomic sequences were also detected within the midrib tissue samples [96].

## Phyllosphere Endophytes as Antagonists of Olive Pathogens

The olive crop is affected by numerous diseases that often lead to severe yield losses [97]. Some of these diseases are considered highly destructive and devastating. One of the most recent and threatening is Olive Quick Decline Syndrome (OQDS), caused by *Xylella fastidiosa* subspecies *pauca*, a xylem-limited phytopathogenic bacterium. In the plant, *X. fastidiosa* behaves similarly to other vascular pathogens, with symptoms progressing from leaf scorch to complete tree collapse [98, 99]. Olive anthracnose, mainly associated with fungal species *Colletotrichum acutatum* and *C. gloeosporioides*, affects fruits negatively impacting the sensory qualities of olive oil and causing considerable production losses [100]. Another widespread and damaging disease is Verticillium wilt of olive (VWO), caused by the soil-borne fungus *Verticillium dahliae*. This pathogen is particularly difficult to manage due to its ability to produce resting structures (microsclerotia) and the existence of two virulent pathotypes (defoliating and non-defoliating), which enhance its dissemination and epidemiology [101].

Currently, disease management relies heavily on copper-based pesticides, which have limited effectiveness and are not compatible with sustainable agricultural practices [24, 102, 103]. The lack of specific, effective treatments underscores the urgent need for alternative, eco-friendly strategies. One promising approach is the use of biological control agents (BCAs), including antagonistic microorganisms that protect plants through direct inhibition of pathogens (Table 1) or by inducing plant defense mechanisms. BCAs can suppress plant pathogens by activating both local and systemic plant defense mechanisms. At the local level, they synthesize and secrete secondary metabolites with defensive functions, compete for nutrients, and colonize plant tissues. BCAs can also activate signaling pathways that induce *induced systemic resistance* (ISR) in the host plant. Beneficial microbes release various compounds, such as lactones, lipopeptides, siderophores, and rhamnolipids, known as elicitors, which are recognized by the plant as signals that enhance defense and resistance against phytopathogens. Defense priming following an initial infection involves the induction of secondary metabolites, oxidative bursts, chromatin modifications, pattern-recognition receptors, antimicrobial compounds, and the synthesis of phytohormones such as ethylene, jasmonic acid, and salicylic acid, all of which contribute to the regulation of induced defense responses [20, 104, 105].

Bacterial and fungal endophytes isolated from resistant Apulian olive cultivars have demonstrated in vitro antagonistic activity against *X. fastidiosa* subsp. *pauca* ST53 [28]. Strains such as *Bacillus subtilis*, *B. pumilus*, *Pantoea agglomerans*, and *Paraconiothyrium brasiliense* significantly inhibited the growth of the pathogen in both dual culture and disc diffusion assays. Notably, *B. subtilis* and *P. brasiliense* successfully colonized the internal tissues of inoculated plants and triggered upregulation of defense-related genes in cultivars ‘Leccino’ and ‘Cima di Mola’ [28]. Similarly, Mourou et al. [106] screened bacterial endophytes isolated from symptomatic and asymptomatic olive trees of cv. ‘Leccino’ and cv. ‘Ogliarola Salentina’, identifying *Paenibacillus naphthalenovorans* as the most effective antagonist, followed by *P. rigui*, *B. subtilis*, *Pseudomonas hibiscicola*, and *B. pumilus*. Cell-free supernatants from *B. subtilis*, *B. pumilus*, and *P. rigui* also showed potent antimicrobial effects, confirming their potential as biocontrol agents.

Several fungal endophytes have also exhibited strong antagonistic effects against *C. acutatum* and *V. dahliae*. Martins et al. [107] identified *Hypocrea lixii* and *Paecilomyces lilacinus* as the most effective in inhibiting mycelial growth of these pathogens, while *Penicillium commune* was able to significantly reduce *C. acutatum* sporulation. In *in planta* assays, *P. commune* (strain CIMO 14FM009) inoculated onto olive branches led to reduced pathogen growth and sporulation, as well as increased release of volatile organic compounds (VOCs) with protective functions [108].

Landum et al. [109] evaluated six fungal endophytes from asymptomatic cv. ‘Galega vulgar’ trees and found that *Nigrospora oryzae* showed strong mutual inhibition with *C. acutatum*. Other isolates such as *Alternaria sp.*, *Epicoccum nigrum*, and *Fusarium sp.* also demonstrated inhibitory effects via direct antagonism and VOC emission. Additional promising isolates included *Chaetomium sp.* and *Diaporthe sp.*, with the latter producing substances that reduced the radial growth of the pathogen. Preto et al. [33] selected *Chondrostereum purpureum* from olives of susceptible cv. ‘Madural’, which inhibited pathogen growth by 30.9% and, although it did not reduce sporulation significantly, it showed potential for future applications. *Aureobasidium pullulans* (strain CIMO 19DM275) also effectively reduced *C. acutatum* growth, sporulation, and infection severity on detached olive fruits [110].

Further evidence of *A. pullulans* efficacy came from Varo et al. [111], who tested four strains from cv. ‘Picual’ and ‘Arbequina’. These strains inhibited mycelial growth of the virulent defoliating pathotype *V. dahliae* V024 by more than 56%. Application of strain AP06 (via foliar spray or irrigation) to olive plants in pathogen-infested soil significantly reduced disease-related mortality. Similarly, *Bacillus amyloliquefaciens*, isolated from European and African olive trees, showed high antagonistic activity against *V. dahliae* V25 in dual culture assays [15]. Finally, *Bacillus licheniformis* Bl_SYLV02R, isolated from wild Sicilian olive twigs and selected for its plant-growth-promoting properties, inhibited more than 40% of the emerging fungal pathogen *Neofusicoccum vitifusiforme* in in vitro tests [29].

**Table 1:** Main endophytes from Olive phyllosphere involved in biotic stress tolerance

Pathogen	Endophytes	Origin of endophyte isolation	Olive host	In vitro inhibition zone (mm) or growth inhibition (%)	Reference
***Xylella fastidiosa***	*Bacillus subtilis*	Sapwood	cv. ‘Leccino’, cv. ‘Ogliarola salentina’, cv. ‘Oliva rossa’	15.2 ± 0.69 (mm)	[28]
	*Bacillus pumilus*	Sapwood	cv. ‘Leccino’, cv. ‘Ogliarola salentina’, cv. ‘Oliva rossa’	8.7 ± 0.47 (mm)	[28]
	*Pantoea agglomerans*	Sapwood	cv. ‘Leccino’, cv. ‘Ogliarola salentina’, cv. ‘Oliva rossa’	11.82 ± 0.42 (mm)	[28]
	*Paraconiothyrium brasiliense*	Sapwood	cv. ‘Leccino’, cv. ‘Ogliarola salentina’, cv. ‘Oliva rossa’	19.28 ± 0.86 (mm)	[28]
	*Paraconiothyrium brasiliense*	Sapwood	cv. ‘Leccino’, cv. ‘Ogliarola salentina’, cv. ‘Oliva rossa’	17.3 ± 0.52 (mm)	[28]
	*Paenibacillus naphthalenovorans*	Twigs	cv. ‘Leccino’	38.6 ± 0.94 (mm)	[106]
	*Paenibacillus rigui*	Twigs	cv. ‘Ogliarola salentina’	21.6 ± 0.47 (mm)	[106]
	*Bacillus subtilis*	Twigs	cv. ‘Leccino’	19 ± 0.4 (mm)	[106]
	*Pseudomonas hibiscicola*	Twigs	cv. ‘Leccino’	15 ± 0.4 (mm)	[106]
	*Bacillus pumilus*	Twigs	cv. ‘Leccino’	9.8 ± 0.23 (mm)	[106]
***Colletotrichum acutatum***	*Hypocrea lixii*	Leaves, branches and roots	cv. ‘Cobrançosa’	50%	[107]
	*Paecilomyces lilacinus*	Leaves, branches and roots	cv. ‘Cobrançosa’	38%	[107]
	*Penicillium commune*	Leaves, branches and roots	cv. ‘Cobrançosa’	n.a.	[107]
	*Penicillium commune*	Twigs	cv. ‘Cobrançosa’	*In plant assay	[108]
	*Nigrospora oryzae*	Leaves of	cv. ‘Galega vulgar’	68%	[109]
	*Alternaria sp.*	Leaves	cv. ‘Galega vulgar’	28%	[109]
	*Epicoccum nigrum*	Leaves	cv. ‘Galega vulgar’	25%	[109]
	*Chaetomium sp.*	Leaves	cv. ‘Galega vulgar’	21%	[109]
	*Fusarium sp.*	Leaves	cv. ‘Galega vulgar’	14%	[109]
	*Diaporthe sp.*	Leaves	cv. ‘Galega vulgar’	12%	[109]
	*Chondrostereum purpureum*	Olive fruits	cv. ‘Madural’	30.9%	[33]
	*Aureobasidium pullulans*	Leaves	cv. ‘Cobrançosa’ and cv. ‘Madural’	39%	[110]
***Verticillium dahliae***	*Hypocrea lixii*	Leaves, branches and roots	cv. ‘Cobrançosa’	55%	[107]
	*Paecilomyces lilacinus*	Leaves, branches and roots	cv. ‘Cobrançosa’	32%	[107]
	*Aureobasidium pullulans* AP06	Leaves	cv. ‘Picual’	57.8%	[111]
	*Aureobasidium pullulans* AP07	Leaves	cv. ‘Arbequina’	56%	[111]
	*Aureobasidium pullulans* AP08	Leaves	cv. ‘Picual’	55.9%	[111]
	*Aureobasidium pullulans* AP09	Leaves	cv. ‘Picual’	58.3%	[111]
	*Bacillus amyloliquefaciens*	Branches and leaves	Different olive cultivars and wild olive trees	n.a.	[15]
***Neofusicoccum vitifusiforme***	*Bacillus licheniformis*	Twigs	Wild olive trees	40.3%	[29]

## Conclusions

Current research on the olive tree microbiota highlights the complexity and ecological significance of associated-endophytic communities. Host-related factors, particularly plant genotype, organ specificity, age, and developmental stage, are key drivers in shaping the diversity and composition of endophytic microbial communities in olive trees. Among these, plant genotype consistently emerges as a major determinant, with both bacterial and fungal communities specific to multiple compartments and geographic regions. Endophyte richness and community composition often differ between leaves and twigs, with some genera uniquely associated with one organ. phyllosphere and carposphere harbor distinct microbiota shaped by their temporal and physiological transitions. Older trees consistently harbor more diverse and established bacterial communities, while seasonal changes influence both bacterial and fungal community dynamics. Geographic location plays an important role in microbial community assembly. Regional differences in soil, climate, and surrounding vegetation strongly influence both bacterial and fungal endophyte profiles, with closer sites typically exhibiting more similar communities. Changes across seasons and local microclimates modulate microbial abundance and diversity, highlighting the dynamic and responsive nature of endophyte assemblages to external environmental cues. Responses to agricultural management appear to be taxa- and cultivar-dependent, indicating complex interactions between host genotype, microbial ecology, and farming inputs. Abiotic and biotic stresses have been shown to alter the balance and structure of endophytic communities, reflecting differential resilience across microbial kingdoms. Certain bacterial and fungal taxa potentially act as keystone microorganisms in suppressing or mitigating infection by olive pathogens. In this context, endophytic microorganisms—particularly cultivable strains of *Bacillus*, *Pantoea*, *Paenibacillus*, *Aureobasidium*, and *Penicillium*—have demonstrated antagonistic activity against major olive pathogens such as *Xylella fastidiosa*, *Colletotrichum spp.*, *Verticillium dahliae*, and *Neofusicoccum* species. These microbial antagonists act via direct competition, inhibition of pathogen growth, or induction of host defense responses, offering promising prospects for their application as biological control agents (BCAs) in integrated and sustainable disease management strategies. Altogether, these findings highlight the need for deeper functional studies to clarify the roles of keystone endophytes in plant–microbe–pathogen interactions and to explore the use of endophytic consortia in integrated management. Future research should aim to unravel the mechanisms by which microbial communities contribute to host health and defense, focusing on their metabolic traits, colonization patterns, and synergistic interactions with the host plant, advancing the development of more resilient, eco-friendly, and productive olive crops.

## Data Availability

No datasets were generated or analysed during the current study.

## References

[1] Schicchi R, Speciale C, Amato F et al (2021) The monumental olive trees as biocultural heritage of Mediterranean landscapes: the case study of Sicily. Sustainability 13:6767:1–17. 10.3390/su13126767

[2] Ladisa G, Calabrese G, Perrino EV (2012) The origin and distribution of olive tree and olive crop. Study on biodiversity in century-old olive groves. Ciheam - lamb

[3] Cardoni M, Mercado-Blanco J (2023) Confronting stresses affecting olive cultivation from the holobiont perspective. Front Plant Sci 14:1261754. 10.3389/fpls.2023.1261754 38023867 10.3389/fpls.2023.1261754 PMC10661416

[4] Fanelli V, Mascio I, Falek W et al (2022) Current status of biodiversity assessment and conservation of wild olive (*Olea Europaea* L. *subsp. Europaea var. sylvestris*). Plants 11:480. 10.3390/plants11040480 35214813 10.3390/plants11040480 PMC8877956

[5] Nteve GM, Kostas S, Polidoros AN et al (2024) Adaptation mechanisms of olive tree under drought stress: the potential of modern omics approaches. Agriculture 14:579. 10.3390/agriculture14040579

[6] Palm ER, Salzano AM, Vergine M et al (2024) Response to salinity stress in four *Olea europaea* L. genotypes: a multidisciplinary approach. Environ Exp Bot 218:105586. 10.1016/j.envexpbot.2023.105586

[7] Hussain K, Fox J-P, Rossi L (2025) Root morphological and anatomical responses of olive tree cultivars ‘Oliana’ and ‘Lecciana’ under salinity stress. Sci Hortic 344:114108. 10.1016/j.scienta.2025.114108

[8] Medeiros de Oliveira E, Hermógenes GM, da Brito L C, et al (2024) Cover crop management systems improves soil quality and mitigate water erosion in tropical Olive orchards. Sci Hortic 330:113092:1–12. 10.1016/j.scienta.2024.113092

[9] Loconsole G, Saponari M, Boscia D et al (2016) Intercepted isolates of *Xylella fastidiosa* in Europe reveal novel genetic diversity. Eur J Plant Pathol 146:85–94. 10.1007/s10658-016-0894-x

[10] Chaudhary P, Agri U, Chaudhary A et al (2022) Endophytes and their potential in biotic stress management and crop production. Front Microbiol 13:933017:1–22. 10.3389/fmicb.2022.933017 10.3389/fmicb.2022.933017 PMC9618965 36325026

[11] Ben zineb A, Barkaoui K, Karray F et al (2022) Olive agroforestry shapes rhizosphere Microbiome networks associated with annual crops and impacts the biomass production under low-rainfed conditions. Front Microbiol 13:977797:1–18. 10.3389/fmicb.2022.977797 10.3389/fmicb.2022.977797 PMC9650424 36386625

[12] Vieira S, Sikorski J, Dietz S et al (2020) Drivers of the composition of active rhizosphere bacterial communities in temperate grasslands. ISME J 14:463–475. 10.1038/s41396-019-0543-4 31659233 10.1038/s41396-019-0543-4 PMC6976627

[13] De Mandal S, Jeon J (2023) Phyllosphere Microbiome in plant health and disease. Plants 12:3481. 10.3390/plants12193481 37836221 10.3390/plants12193481 PMC10575124

[14] Abdelfattah A, Li Destri Nicosia MG, Cacciola SO et al (2015) Metabarcoding analysis of fungal diversity in the phyllosphere and Carposphere of Olive (*Olea europaea*). PLoS ONE 10:1–19. 10.1371/journal.pone.0131069 10.1371/journal.pone.0131069 PMC4489200 26132745

[15] Müller H, Berg C, Landa BB et al (2015) Plant genotype-specific archaeal and bacterial endophytes but similar *Bacillus* antagonists colonize mediterranean Olive trees. Front Microbiol 6:1–9. 10.3389/fmicb.2015.00138 25784898 10.3389/fmicb.2015.00138 PMC4347506

[16] Costa D, Fernandes T, Martins F et al (2021) Illuminating *Olea Europaea* L. endophyte fungal community. Microbiol Res 245:1–10. 10.1016/j.micres.2020.126693 10.1016/j.micres.2020.126693 33482404

[17] de Oliveira AA, Ramalho M, de Moreau O CS, et al (2022) Exploring the diversity and potential interactions of bacterial and fungal endophytes associated with different cultivars of Olive (*Olea europaea*) in Brazil. Microbiol Res 263:1–17. 10.1016/j.micres.2022.127128 10.1016/j.micres.2022.127128 35868260

[18] Malacrinò A, Mosca S, Li Destri Nicosia MG et al (2022) Plant genotype shapes the bacterial Microbiome of Fruits, Leaves, and soil in Olive plants. Plants 11:1–9. 10.3390/plants11050613 10.3390/plants11050613 PMC8912820 35270082

[19] Crucitti D, Sonnessa M, Carimi F et al (2025) Endophytic microbiota diversity in the phyllosphere of Sicilian Olive trees across growth phases and farming systems. Curr Plant Biol 43:100510:1–15. 10.1016/j.cpb.2025.100510

[20] Gómez-Lama Cabanás C, Mercado-Blanco J (2020) What Determines Successful Colonization and Expression of Biocontrol Traits at the Belowground Level? In: De Cal A, Melgarejo P, Magan N (eds) Progress in Biological Control. Springer Nature Switzerland AG, pp 31–46

[21] Dias MC, Silva S, Galhano C, Lorenzo P (2024) Olive tree belowground microbiota: plant growth-promoting bacteria and fungi. Plants 13:1848. 10.3390/plants13131848 38999688 10.3390/plants13131848 PMC11244348

[22] Martins F, Pereira JA, Bota P et al (2016) Fungal endophyte communities in above- and belowground olive tree organs and the effect of season and geographic location on their structures. Fungal Ecol 20:193–201. 10.1016/j.funeco.2016.01.005

[23] Gomes T, Pereira JA, Benhadi J et al (2018) Endophytic and epiphytic phyllosphere fungal communities are shaped by different environmental factors in a mediterranean ecosystem. Microb Ecol 76:668–679. 10.1007/s00248-018-1161-9 29500493 10.1007/s00248-018-1161-9

[24] Materatski P, Varanda C, Carvalho T et al (2019) Spatial and temporal variation of fungal endophytic richness and diversity associated to the phyllosphere of olive cultivars. Fungal Biol 123:66–76. 10.1016/j.funbio.2018.11.004 30654959 10.1016/j.funbio.2018.11.004

[25] Mina D, Pereira JA, Lino-Neto T, Baptista P (2020) Epiphytic and endophytic bacteria on olive tree phyllosphere: exploring tissue and cultivar effect. Microb Ecol 80:145–157. 10.1007/s00248-020-01488-8 31965223 10.1007/s00248-020-01488-8

[26] Anguita-Maeso M, Haro C, Montes-Borrego M et al (2021) Metabolomic, ionomic and microbial characterization of Olive xylem Sap reveals differences according to plant age and genotype. Agronomy 11:1179:1–21. 10.3390/agronomy11061179

[27] Martins F, Mina D, Pereira JA, Baptista P (2021) Endophytic fungal community structure in olive orchards with high and low incidence of olive anthracnose. Sci Rep 11(1):68g. 10.1038/s41598-020-79962-z 33436767 10.1038/s41598-020-79962-z PMC7804420

[28] Hanani A, Valentini F, Sanzani SM et al (2022) Community analysis of culturable sapwood endophytes from Apulian Olive varieties with different susceptibility to *Xylella fastidiosa*. Agronomy 12:9:1–16. 10.3390/agronomy12010009

[29] Crucitti D, Barone S, Navarro-Torre S et al (2025) Endophytic diversity in Sicilian Olive Trees: identifying optimal conditions for a functional microbial collection. Microorganisms 13:1502. 10.3390/microorganisms13071502 40732010 10.3390/microorganisms13071502 PMC12298726

[30] Caliz J, Montes-Borrego M, Triadó-Margarit X et al (2015) Influence of edaphic, climatic, and agronomic factors on the composition and abundance of nitrifying microorganisms in the rhizosphere of commercial olive crops. PLoS One. 10.1371/journal.pone.0125787 25950678 10.1371/journal.pone.0125787 PMC4423868

[31] Giampetruzzi A, Baptista P, Morelli M et al (2020) Differences in the endophytic microbiome of olive cultivars infected by *Xylella fastidiosa* across seasons. Pathogens 9(9):723. 10.3390/pathogens9090723 32887278 10.3390/pathogens9090723 PMC7558191

[32] Chow C, Padda KP, Puri A, Chanway CP (2022) An archaic approach to a modern issue: endophytic archaea for sustainable agriculture. Curr Microbiol 79:322. 10.1007/s00284-022-03016-y 36125558 10.1007/s00284-022-03016-y

[33] Preto G, Martins F, Pereira JA, Baptista P (2017) Fungal community in Olive fruits of cultivars with different susceptibilities to anthracnose and selection of isolates to be used as biocontrol agents. Biol Control 110:1–9. 10.1016/j.biocontrol.2017.03.011

[34] Ngubane NP, Dreyer LL, Slippers B et al (2023) Decreased diversity and connectivity of endophytic fungal assemblages within cultivated European olive trees compared to their native African counterpart. Fungal Ecol 65:101261. 10.1016/j.funeco.2023.101261

[35] Li Y, Wu X, Chen T et al (2018) Plant phenotypic traits eventually shape its microbiota: a common garden test. Front Microbiol 9:2479. 10.3389/fmicb.2018.02479 30459725 10.3389/fmicb.2018.02479 PMC6232875

[36] Laforest-Lapointe I, Messier C, Kembel SW (2016) Host species identity, site and time drive temperate tree phyllosphere bacterial community structure. Microbiome 4:27. 10.1186/s40168-016-0174-1 27316353 10.1186/s40168-016-0174-1 PMC4912770

[37] Unterseher M, Siddique AB, Brachmann A, Peršoh D (2016) Diversity and composition of the leaf mycobiome of beech (*Fagus sylvatica*) are affected by local habitat conditions and leaf biochemistry. PLoS One 11(4):e0152878. 10.1371/journal.pone.0152878 27078859 10.1371/journal.pone.0152878 PMC4831807

[38] Anderson HM, Cagle GA, Majumder ELW et al (2024) Root exudation and rhizosphere microbial assembly are influenced by novel plant trait diversity in carrot genotypes. Soil Biol Biochem 197:109516. 10.1016/J.SOILBIO.2024.109516

[39] Vita F, Sabbatini L, Sillo F et al (2022) Salt stress in olive tree shapes resident endophytic microbiota. Front Plant Sci 13:9923951–9923919. 10.3389/fpls.2022.992395 10.3389/fpls.2022.992395 PMC9556989 36247634

[40] Wentzien NM, Fernández-González AJ, Valverde-Corredor A et al (2024) Pitting the olive seed microbiome. Environ Microbiol 19:17. 10.1186/s40793-024-00560-x 10.1186/s40793-024-00560-x PMC10943921 38491515

[41] Zhang J, Liu W, Bu J et al (2023) Host genetics regulate the plant microbiome. Curr Opin Microbiol 72:102268. 10.1016/J.MIB.2023.102268 36708613 10.1016/j.mib.2023.102268

[42] Barnes CJ, Bahram M, Nicolaisen M et al (2025) Microbiome selection and evolution within wild and domesticated plants. Trends Microbiol 33(4):447–458 39701859 10.1016/j.tim.2024.11.011

[43] Dastogeer KMG, Tumpa FH, Sultana A et al (2020) Plant microbiome–an account of the factors that shape community composition and diversity. Curr Plant Biol 23:100161:1–9. 10.1016/j.cpb.2020.100161

[44] Kandel SL, Joubert PM, Doty SL (2017) Bacterial endophyte colonization and distribution within plants. Microorganisms 5:77. 10.3390/microorganisms5040077 29186821 10.3390/microorganisms5040077 PMC5748586

[45] González-Teuber M, Palma-Onetto V, Aguilera-Sammaritano J, Mithöfer A (2021) Roles of leaf functional traits in fungal endophyte colonization: potential implications for host–pathogen interactions. J Ecol 109(12):3972–3987. 10.1111/1365-2745.13678

[46] Romero C, García A, Medina E et al (2010) Triterpenic acids in table olives. Food Chem 118:670–674. 10.1016/j.foodchem.2009.05.037

[47] Ramírez EM, Brenes M, Romero C, Medina E (2022) Chemical and enzymatic characterization of leaves from Spanish table Olive cultivars. Foods 11:3879:1–13. 10.3390/foods11233879 10.3390/foods11233879 PMC9738326 36496690

[48] Petrini O, Sieber TN, Toti L, Viret O (1992) Ecology, metabolite production, and substrate utilization in endophytic fungi. Nat Toxins 1:185–196 1344919 10.1002/nt.2620010306

[49] Compant S, Clément C, Sessitsch A (2010) Plant growth-promoting bacteria in the rhizo- and endosphere of plants: their role, colonization, mechanisms involved and prospects for utilization. Soil Biol Biochem 42:669–678. 10.1016/j.soilbio.2009.11.024

[50] Anguita-Maeso M, Olivares-García C, Haro C et al (2020) Culture-dependent and culture-independent characterization of the Olive xylem microbiota: effect of sap extraction methods. Front Plant Sci 10:1–14. 10.3389/fpls.2019.01708 10.3389/fpls.2019.01708 PMC6988092 32038682

[51] Anguita-Maeso M, Navas-Cortés JA, Landa BB (2023) Insights into the Methodological, biotic and abiotic factors influencing the characterization of Xylem-Inhabiting microbial communities of Olive trees. Plants 12:912:1–20. 10.3390/plants12040912 10.3390/plants12040912 PMC9967459 36840260

[52] Harrison JG, Griffin EA (2020) The diversity and distribution of endophytes across biomes, plant phylogeny and host tissues: how far have we come and where do we go from here? Environ Microbiol 22:2107–2123. 10.1111/1462-2920.14968 32115818 10.1111/1462-2920.14968 PMC7679042

[53] Arnold AE, Mejìa LC, Kyllo D et al (2003) Fungal endophytes limit pathogen damage in a tropical tree. Proc Natl Acad Sci USA 100(26):15649–15654. 10.1073/pnas.2533483100 14671327 10.1073/pnas.2533483100 PMC307622

[54] Truyens S, Weyens N, Cuypers A, Vangronsveld J (2015) Bacterial seed endophytes: genera, vertical transmission and interaction with plants. Environ Microbiol Rep 7(1):40–50. 10.1111/1758-2229.12181

[55] Nelson EB (2018) The seed microbiome: origins, interactions, and impacts. Plant Soil 422:7–34. 10.1007/s11104-017-3289-7

[56] Higgins KL, Arnold AE, Miadlikowska J et al (2007) Phylogenetic relationships, host affinity, and geographic structure of boreal and arctic endophytes from three major plant lineages. Mol Phylogenet Evol 42:543–555. 10.1016/j.ympev.2006.07.012 17005421 10.1016/j.ympev.2006.07.012

[57] Campisano A, Albanese D, Yousaf S et al (2017) Temperature drives the assembly of endophytic communities’ seasonal succession. Environ Microbiol 19(8):3353–3364. 10.1111/1462-2920.13843 28654220 10.1111/1462-2920.13843

[58] Argiroff WA, Carrell AA, Klingeman DM et al (2024) Seasonality and longer-term development generate Temporal dynamics in the Populus Microbiome. mSystems 9(3):e0088623. 10.1128/msystems.00886-23 38421171 10.1128/msystems.00886-23 PMC10949431

[59] Drossopoulos JB, Niavis CA (1988) Seasonal changes of the metabolites in the Leaves, bark and xylem tissues of Olive tree (*Olea Europaea* L) I. nitrogenous compounds. Ann Bot 62(3):313–320

[60] Baktir I, Ulger S, Kaynak L, Himelrick DG (2004) Relationship of seasonal changes in endogenous plant hormones and alternate bearing of Olive trees. HortScience 39(5):987–990. 10.21273/hortsci.39.5.987

[61] Divon HH, Fluhr R (2007) Nutrition acquisition strategies during fungal infection of plants. FEMS Microbiol Lett 266:65–74. 10.1111/j.1574-6968.2006.00504.x 17083369 10.1111/j.1574-6968.2006.00504.x

[62] López-Ráez JA, Pozo MJ (2013) Chemical Signalling in the Arbuscular Mycorrhizal Symbiosis: Biotechnological Applications. In: Aroca R (ed) Symbiotic Endophytes, Soil Biology. Springer-Verlag Berlin Heidelberg, pp 215–232

[63] Goh DM, Cosme M, Kisiala AB et al (2019) A stimulatory role for cytokinin in the arbuscular mycorrhizal symbiosis of pea. Front Plant Sci 10:262. 10.3389/fpls.2019.00262 30915091 10.3389/fpls.2019.00262 PMC6423060

[64] Talley SM, Coley PD, Kursar TA (2002) The effects of weather on fungal abundance and richness among 25 communities in the intermountain West. BMC Ecol 2:7:1–11 12079496 10.1186/1472-6785-2-7 PMC117440

[65] Martinez-Alvarez P, Martin-Garcia J, Rodriguez-Ceinos S, Diez JJ (2012) Monitoring endophyte populations in pine plantations and native oak forests in Northern Spain. Syst 21(3):373–382. 10.5424/fs/2012213-02254

[66] Kremen C, Miles A (2012) Ecosystem services in biologically diversified versus conventional farming systems: benefits, externalities, and trade-offs. Ecology and Society 17(4):40. 10.5751/ES-05035-170440

[67] Beltran-Garcia MJ, Martínez-Rodríguez A, Olmos-Arriaga I et al (2021) Nitrogen fertilization and stress factors drive shifts in microbial diversity in soils and plants. Symbiosis 84:379–390. 10.1007/s13199-021-00787-z

[68] Khatri S, Dubey S, Shivay YS et al (2023) Organic farming induces changes in bacterial community and disease suppressiveness against fungal phytopathogens. Appl Soil Ecol 181:104658. 10.1016/j.apsoil.2022.104658

[69] Chen H, Xia Q, Yang T, Shi W (2018) Eighteen-year farming management moderately shapes the soil microbial community structure but promotes habitat-specific taxa. Front Microbiol 9:1776. 10.3389/fmicb.2018.01776 30116234 10.3389/fmicb.2018.01776 PMC6083213

[70] Oliveira MCO, Alves A, Ragonezi C et al (2024) Organic farming enhances diversity and recruits beneficial soil fungal groups in traditional banana plantations. Microorganisms 12:2372. 10.3390/microorganisms12112372 39597760 10.3390/microorganisms12112372 PMC11596534

[71] Nam B, Lee HJ, Choi YJ (2023) Organic farming allows balanced fungal and oomycetes communities. Microorganisms 11:1307. 10.3390/microorganisms11051307 37317281 10.3390/microorganisms11051307 PMC10221069

[72] Díaz-Rodríguez AM, Parra Cota FI, Cira Chávez LA et al (2025) Microbial inoculants in sustainable agriculture: advancements, challenges, and future directions. Plants 14:191. 10.3390/plants14020191 39861545 10.3390/plants14020191 PMC11768969

[73] Rungjindamai N, Jones EBG (2024) Why are there so few Basidiomycota and basal fungi as endophytes? A review. J Fungi 10:67. 10.3390/jof10010067 10.3390/jof10010067 PMC10820240 38248976

[74] Pascazio S, Crecchio C, Ricciuti P et al (2015) Phyllosphere and Carposphere bacterial communities in Olive plants subjected to different cultural practices. Int J Plant Biology 6:6011:1–19. 10.4081/pb.2015.6011

[75] Marzouk T, Kaushik N, Chaouachi M et al (2022) Endophytic microbes modulate plant responses to abiotic stresses: a review. J Oasis Agric Sustainable Dev 4(3):62–85. 10.56027/JOASD172022

[76] Rath KM, Maheshwari A, Rousk J (2019) Linking microbial community structure to trait distributions and functions using salinity as an environmental filter. MBio 10:e01607–e01619. 10.1128/mBio.01607-19 31337729 10.1128/mBio.01607-19 PMC6650560

[77] Moreira V, Carbone MJ, Mondino P, Alaniz S (2023) *Colletotrichum* infections during flower development and fruit ripening in four Olive cultivars. Phytopathol Mediterr 62(1):35–46. 10.36253/phyto-14087

[78] Varanda CMR, Materatski P, Landum M et al (2019) Fungal communities associated with Peacock and cercospora leaf spots in Olive. Plants 8(6):169:1–14. 10.3390/plants8060169 10.3390/plants8060169 PMC6630884 31212781

[79] Vergine M, Meyer JB, Cardinale M et al (2020) The *Xylella fastidiosa*-resistant Olive cultivar leccino has stable endophytic microbiota during the Olive quick decline syndrome (OQDS). Pathogens 9:35:1–23. 10.3390/pathogens9010035 10.3390/pathogens9010035 PMC7168594 31906093

[80] Lacava PT, Li W, Luiz W et al (2007) The endophyte *Curtobacterium flaccumfaciens* reduces symptoms caused by *Xylella fastidiosa* in *Catharanthus roseus*. J Microbiol 45(5):388–393 17978797

[81] Zicca S, De Bellis P, Masiello M et al (2020) Antagonistic activity of Olive endophytic bacteria and of *Bacillus* spp. Strains against *Xylella fastidiosa*. Microbiol Res 236:126467:1–7. 10.1016/J.MICRES.2020.126467 10.1016/j.micres.2020.126467 32248049

[82] Vergine M, Vita F, Casati P et al (2024) Characterization of the olive endophytic community in genotypes displaying a contrasting response to *Xylella fastidiosa*. BMC Plant Biol 24:1–17. 10.1186/s12870-024-04980-2 38664617 10.1186/s12870-024-04980-2 PMC11044560

[83] Vergine M, Vita F, De Pascali M et al (2025) How *Xylella fastidiosa* subsp. *Pauca* influences endophytic communities and plant physiology in resistant and susceptible Olive tree cultivars. Plant Stress 17:100924:1–21. 10.1016/j.stress.2025.100924

[84] Zhou X, Wang J, Liu F et al (2022) Cross-kingdom synthetic microbiota supports tomato suppression of fusarium wilt disease. Nat Commun 13:7890. 10.1038/s41467-022-35452-6 36550095 10.1038/s41467-022-35452-6 PMC9780251

[85] Al Raish SM, Sourani OM, Abu-Elsaoud AM (2025) Plant growth-promoting microorganisms as biocontrol agents: mechanisms, challenges, and future prospects. Appl Microbiol 5:44. 10.3390/applmicrobiol5020044

[86] Gomes T, Pereira JA, Lino-Neto T et al (2019) Bacterial disease induced changes in fungal communities of Olive tree twigs depend on host genotype. Sci Rep 9:5882:1–10. 10.1038/s41598-019-42391-8 30971758 10.1038/s41598-019-42391-8 PMC6458152

[87] Pascale A, Proietti S, Pantelides IS, Stringlis IA (2020) Modulation of the root microbiome by plant molecules: the basis for targeted disease suppression and plant growth promotion. Front Plant Sci. 10.3389/fpls.2019.01741 32038698 10.3389/fpls.2019.01741 PMC6992662

[88] Wang Y, Dai CC (2011) Endophytes: a potential resource for biosynthesis, biotransformation, and biodegradation. Ann Microbiol 61:207–215. 10.1007/s13213-010-0120-6

[89] Pieterse CMJ, Zamioudis C, Berendsen RL et al (2014) Induced systemic resistance by beneficial microbes. Annu Rev Phytopathol 52:347–375. 10.1146/annurev-phyto-082712-102340 24906124 10.1146/annurev-phyto-082712-102340

[90] Wang C, Xiao Z, Cao Z et al (2025) Fungal and bacterial pathogenic co-infections mainly lead to the assembly of microbial community in tobacco stems. Open Life Sci 20:20251103. 10.1515/biol-2025-1103 40989583 10.1515/biol-2025-1103 PMC12451430

[91] Flores-Nunez VM, Stukenbrock EH (2024) The impact of filamentous plant pathogens on the host microbiota. BMC Biol 22:175. 10.1186/s12915-024-01965-3 39148076 10.1186/s12915-024-01965-3 PMC11328434

[92] Licciardello G, Mosca A, Di Silvestro S et al (2023) Cultivar susceptibility to Olive knot disease and association with endophytic microbiota community. Agronomy 13:468:1–14. 10.3390/agronomy13020468

[93] Malacrinò A, Schena L, Campolo O et al (2017) A metabarcoding survey on the fungal microbiota associated to the Olive fruit fly. Microb Ecol 73:677–684. 10.1007/s00248-016-0864-z 27687872 10.1007/s00248-016-0864-z

[94] Gharsallah H, Ksentini I, Frikha-Gargouri O et al (2023) Exploring bacterial and fungal biodiversity in eight mediterranean Olive orchards (*Olea europaea* L.) in Tunisia. Microorganisms 11:1086. 10.3390/microorganisms11041086 37110509 10.3390/microorganisms11041086 PMC10145363

[95] Abdelfattah A, Ruano-Rosa D, Cacciola SO et al (2018) Impact of *Bactrocera oleae* on the fungal microbiota of ripe olive drupes. PLoS One 13(11):e0199403. 10.1371/journal.pone.0199403 30496186 10.1371/journal.pone.0199403 PMC6264826

[96] Azizpour N, Nematollahi S, Khakvar R et al (2022) Identification of endophytic microbiota of Phytoplasma-infected Russian olive trees “Elaeagnus angustifolia L.” in the Northwest of Iran. Forests 13:1684. 10.3390/f13101684

[97] Alarcón Roldàn R, Álvarez B, Bernardo U et al (2020) EIP-AGRI Focus Group Pests and diseases of the olive tree: Final report. Available online: https://ec.europa.eu/eip/agriculture/en/publications/eip-agri-focus-group-pests-and-diseases-olive-tree-0.html (accessed on 21 June 2025)

[98] Scortichini M (2022) The epidemiology and control of “Olive Quick Decline Syndrome” in Salento (Apulia, Italy). Agronomy 12:2475:1–26. 10.3390/agronomy12102475

[99] Scortichini M, Manetti G, Brunetti A et al (2023) *Xylella fastidiosa* subsp. *pauca*, *Neofusicoccum* spp. and the decline of olive trees in Salento (Apulia, Italy): comparison of symptoms, possible interactions, certainties and doubts. Plants 12:3593. 10.3390/plants12203593 37896056 10.3390/plants12203593 PMC10609838

[100] Cacciola SO, Faedda R, Sinatra F et al (2012) Olive anthracnose. J Plant Pathol 94(1):29–44

[101] Montes-Osuna N, Mercado-Blanco J (2020) Verticillium wilt of olive and its control: what did we learn during the last decade? Plants 9(735):1–31. 10.3390/plants9060735 10.3390/plants9060735 PMC7356185 32545292

[102] Bragard C, Dehnen-Schmutz K, Di Serio F et al (2019) Update of the scientific opinion on the risks to plant health posed by *Xylella fastidiosa* in the EU territory. EFSA J 17(5):5665. 10.2903/j.efsa.2019.5665 10.2903/j.efsa.2019.5665 PMC7009223 32626299

[103] López-Moral A, Agustì-Brisach C, Trapero A (2021) Plant biostimulants: new insights into the biological control of Verticillium wilt of olive. Front Plant Sci 12:662178. 10.3389/fpls.2021.662178 34093620 10.3389/fpls.2021.662178 PMC8172626

[104] Dimopoulou A, Theologidis I, Liebmann B et al (2019) *Bacillus amyloliquefaciens* MBI600 differentially induces tomato defense signaling pathways depending on plant part and dose of application. Sci Rep 9:19120. 10.1038/s41598-019-55645-2 31836790 10.1038/s41598-019-55645-2 PMC6910970

[105] Kour D, Negi R, Khan SS et al (2024) Microbes mediated induced systemic response in plants: a review. Plant Stress 11:100334. 10.1016/j.stress.2023.100334

[106] Mourou M, Hanani A, D’onghia AM et al (2022) Antagonism and antimicrobial capacity of epiphytic and endophytic bacteria against the phytopathogen *Xylella fastidiosa*. Agronomy 12:1266:1–12. 10.3390/agronomy12061266

[107] Martins F, Pereira J, Bento A, Baptista P (2013) Potentialities of endophytic fungi of olive tree as biological control agents against *Colletotrichum acutatum* and *Verticillium dahliae*. In: Schneider C, Leifert C, Feldmann F (eds) 5th International Symposium on Plant Protection and Plant Health in Europe. DPG, Berlin-Dahlem, Germany, p 190

[108] Silva S, da Costa H, Lopes T et al (2023) Potential of the endophyte *Penicillium commune* in the control of Olive anthracnose via induction of antifungal volatiles in host plant. Biol Control 187:105373:1–12. 10.1016/j.biocontrol.2023.105373

[109] Landum MC, Félix MdoR, Alho J et al (2016) Antagonistic activity of fungi of *Olea europaea* L. against *Colletotrichum acutatum*. Microbiol Res 183:100–108. 10.1016/j.micres.2015.12.001 26805623 10.1016/j.micres.2015.12.001

[110] Sdiri Y, Lopes T, Rodrigues N et al (2022) Biocontrol ability and production of volatile organic compounds as a potential mechanism of action of Olive endophytes against *Colletotrichum acutatum*. Microorganisms 10:571:1–17. 10.3390/microorganisms10030571 10.3390/microorganisms10030571 PMC8954755 35336146

[111] Varo A, Raya-Ortega MC, Trapero A (2016) Selection and evaluation of micro-organisms for biocontrol of *Verticillium dahliae* in olive. J Appl Microbiol 121:767–777. 10.1111/jam.13199 27277382 10.1111/jam.13199

